# Bidirectional Association Between Frailty and Cardiac Structure and Function: The Atherosclerosis Risk in Communities Study

**DOI:** 10.1161/JAHA.122.029458

**Published:** 2023-07-31

**Authors:** Diego Ramonfaur, Hicham Skali, Brian Claggett, B. Gwen Windham, Priya Palta, Dalane Kitzman, Chiadi Ndumele, Suma Konety, Amil M. Shah

**Affiliations:** ^1^ Department of Medicine, Cardiovascular Medicine Division, Brigham and Women’s Hospital Boston MA; ^2^ The MIND Center University of Mississippi Medical Center Jackson MS; ^3^ Division of General Medicine, Departments of Medicine and Epidemiology Columbia University Irving Medical Center New York NY; ^4^ Division of Cardiology, Department of Medicine Johns Hopkins University Baltimore MD; ^5^ Division of Cardiology Department of Medicine Johns Hopkins University School of Medicine Baltimore MD; ^6^ Johns Hopkins Ciccarone Center for the Prevention of Cardiovascular Disease Division of Cardiology Department of Medicine Johns Hopkins University School of Medicine Baltimore MD; ^7^ University of Minnesota Minneapolis MN

**Keywords:** echocardiography, epidemiology, frailty, LV function, Aging, Epidemiology, Risk Factors, Ultrasound, Heart Failure

## Abstract

**Background:**

Frailty and heart failure frequently coexist in late life. Limited data exist regarding the longitudinal associations of frailty and subclinical cardiac dysfunction. We aim to quantify the association of frailty with longitudinal changes in cardiac function and of cardiac function with progression in frailty status in older adults.

**Methods and Results:**

Participants in the Atherosclerosis Risk in Communities cohort underwent frailty assessments at Visit 5 (V5; 2011–2013), V6 (2016–2017), and V7 (2018–2019), and echocardiographic assessments at V5 and V7. We assessed the association between frailty status at V5 and changes in frailty status from V5 to V7 and changes in cardiac function over 6 years. We then evaluated the association of cardiac function measured at Visit 5 with progression in frailty status over 4 years. Multivariable regression models adjusted for demographics and comorbidities. Among 2574 participants free of heart failure at V5 and V7 (age 74±4 years at V5 and 81±4 years at V7), 3% (n=83) were frail. Frailty at V5 was associated with greater left atrial volume index and E/e' ratio at V5 and 7. Participants who transitioned from robust at V5 to frail at V7 demonstrated greater increases in left ventricular mass index, left atrial volume index, and E/e' over the same period. Among 1648 robust participants at Visit 5, greater left ventricular mass index and mean wall thickness, lower tissue Doppler imaging e', and higher E/e' ratio at Visit 5 were associated with progression in frailty status.

**Conclusions:**

Among robust, older adults free of heart failure, progression in frailty and subclinical left ventricular remodeling and diastolic dysfunction are interrelated.

**Nonstandard Abbreviations and Acronyms**
ARIC: Atherosclerosis Risk in Communities
LAVi: left atrial volume index
LVMi: left ventricular mass index

**Clinical Perspective**

**What Is New?**

- Among older adults free of heart failure, progression in frailty and in subclinical left ventricular remodeling and diastolic dysfunction are interrelated and possibly bidirectional, supporting a common pathobiology.
- Greater left ventricular mass and higher left ventricular filling pressure are associated with greater progression in frailty status, whereas transitioning from robust to prefrail or frail is associated with greater concomitant increases in left ventricular mass and filling pressure.

**What Are the Clinical Implications?**

- As both frailty status and cardiac structure and function are interrelated and modifiable, interventions to modify one may exert beneficial effects on the other.

Increasing life expectancy is expected to result in greater numbers of older individuals and an increase in the attendant challenges of late life.^1^ Older individuals are at heightened risk of frailty, a geriatric clinical syndrome characterized by loss of homeostatic reserves and increased vulnerability to internal and external stressors.^2^ Frailty is a reversible,^3^ albeit burdensome condition, affecting ≈15% of individuals 65 years or older.^4^ Older individuals are also at a heightened risk for heart failure (HF), and aging—independent of cardiovascular risk factors—^5^, ^6^ is associated with alterations in cardiac structure and function that underlie the development of HF.^7^, ^8^, ^9^ Frailty is common in patients with prevalent HF,^10^, ^11^ among whom it associates with higher risk of adverse outcomes.^12^ In addition, frail individuals are at higher risk of cardiovascular morbidity and mortality, including incident HF,^13^ compared with robust individuals.^14^, ^15^ Cross‐sectional studies demonstrate subclinical alterations in cardiac structure and function in frail individuals.^16^ In addition to being common in late life, both frailty and cardiac dysfunction may share systemic inflammation as a common pathobiology.^17^, ^18^, ^19^ However, the temporal relationship between alterations in cardiac function and the development of frailty is unclear.

The objective of this study is to investigate the bidirectional associations between frailty and cardiac dysfunction among older adults in the community. Leveraging longitudinal cardiac and echocardiographic phenotyping in the community‐based ARIC (Atherosclerosis Risk in Communities) study, we first evaluated the association of frailty—and progression in frailty status—with subsequent changes in cardiac structure and function over 6 years in late life. We then assessed the extent to which measures of cardiac structure and function at a single time point predict the progression to frailty over 4 years in late life.

## Methods

The ARIC study was reviewed and approved by the institutional review board of each participating site. Written informed consent was obtained from all participants. Anonymized data from the ARIC study have been made publicly available at the National Heart, Lung, and Blood Institute Biologic Specimen and Data Repository Information Coordinating Center and can be accessed at https://biolincc.nhlbi.nih.gov/studies/aric/. The code used for statistical analyses in this study is available from the corresponding author upon reasonable request.

### Study Population

**Figure 1:**
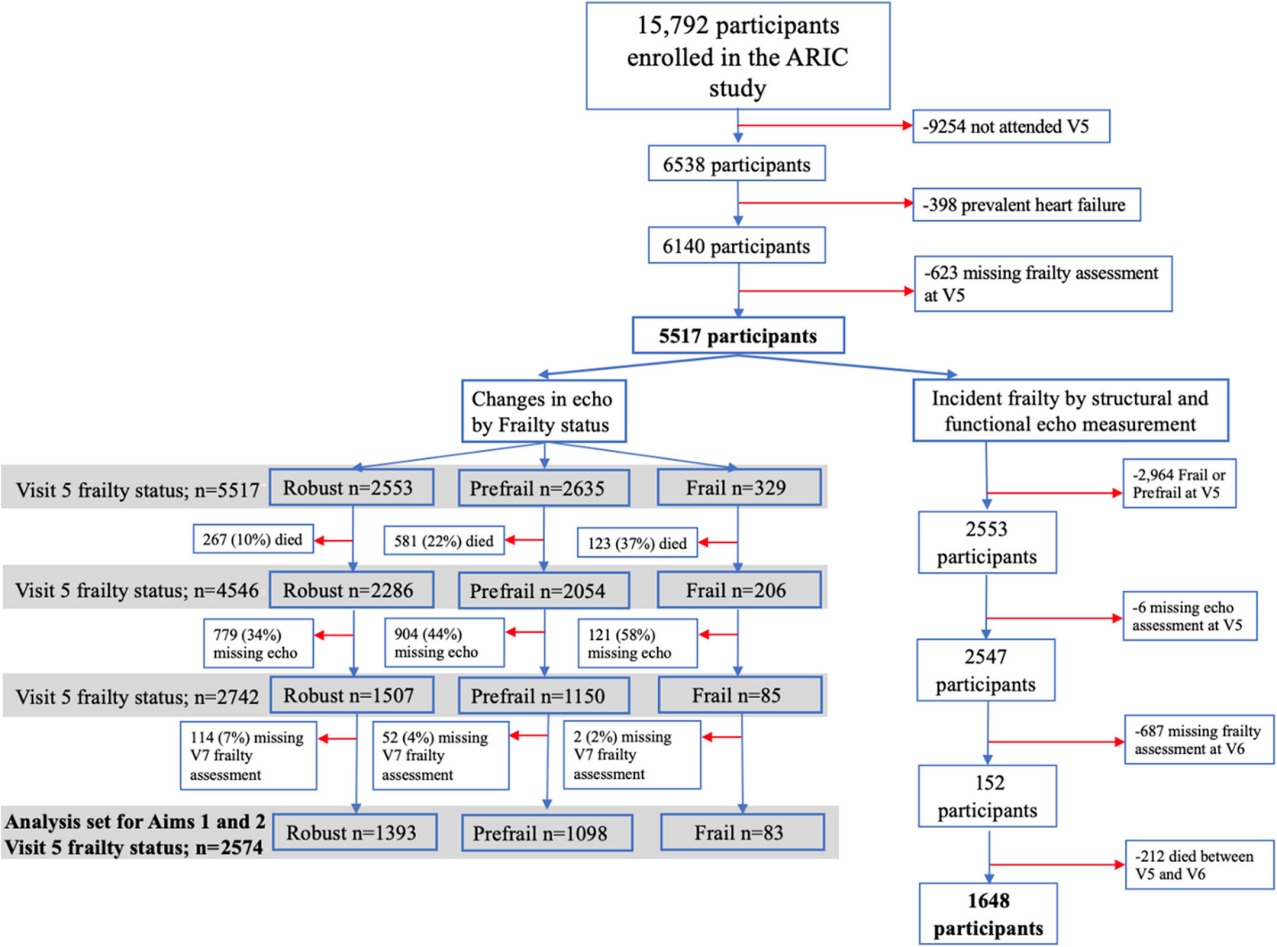
CONSORT diagram summarizing the study population of each analysis. Red arrows indicate that the subset of participants was excluded from analysis. ARIC indicates Atherosclerosis Risk in Communities; and CONSORT, Consolidated Standards of Reporting Trials.

The ARIC study design and procedures have been previously described.^20^, ^21^ ARIC is an ongoing, prospective observational cohort study that originally enrolled 15 792 participants aged 45 to 64 years recruited between 1987 and 1989 (Visit 1) from 4 communities in the United States: Forsyth County, North Carolina; Jackson, Mississippi; Minneapolis, Minnesota; and Washington County, Maryland. Three subsequent study visits occurred between 1989 and 1998, followed by Visit 5 (2011–2013), Visit 6 (2016–2017), and Visit 7 (2018–2019). Standardized frailty assessment and echocardiography were first performed in ARIC at study visit 5 (2011–2013). Frailty assessment was repeated at Visit 6 and Visit 7, and echocardiography was repeated at Visit 7. Among the 10 040 surviving participants by the end of Visit 5, 6538 participants attended that visit. We excluded 398 participants with prevalent HF at Visit 5 and 623 additional participants with a missing frailty assessment. A total of 5517 participants were included in the study before analysis‐specific inclusion criteria were applied. To evaluate the association of frailty and progression in frailty status with changes in cardiac structure and function over 6 years in late life, participants with concomitant echocardiographic and frailty assessment at both Visits 5 and 7 were included (n=2574; Visit 6 frailty data were not included in this analysis). To assess the association of measures of cardiac structure and function at a single time point with risk of incident frailty over 4 years, participants who were robust at Visit 5, underwent echocardiography at Visit 5, and had frailty assessment at Visit 6 were included (n=1648; Visit 7 frailty and echocardiographic data were not included in this analysis to minimize participant attrition). The complete Consolidated Standards of Reporting Trials diagram of the study sample is displayed in Figure 1.

### Frailty Assessment

Frailty was defined using the Fried criteria, a previously validated measure^22^ implemented in several other cohort studies^2^, ^16^, ^23^ that consists of 5 binary criteria: (1) low energy expenditure, based on sex‐specific 20th percentile rank of the Baecke leisure sports activity index; (2) low gait speed, based on the bottom sex and height‐specific quintile of time to walk 15 feet; (3) lack of energy (exhaustion), defined as present if the participant responded “some of the time” or “most of the time” to either of the following questions: “I felt everything I did was an effort” or “I could not get going”; (4) low grip strength, based on the bottom sex‐ and body mass index‐specific grip strength quintile; and (5) unintentional weight loss, defined as 10% unintentional weight loss between Visits 4 (1996–1998) and 5 or body mass index <18.5 kg/m^2^ at Visit 5. Frailty was defined by the presence of at least 3 of these criteria. Participants with 1 or 2 criteria were classified as prefrail, and participants with 0 criteria were classified as robust.

### Echocardiography

Detailed protocols and procedures for echocardiography at ARIC Visit 5, including reproducibility metrics, have been previously described in detail, and equivalent procedures were employed at study Visit 7.^24^ Briefly, all studies were performed by trained study sonographers certified in performance of the study‐specific imaging protocol using uniform echocardiography equipment. Quantitative analysis at both visits was performed by the same central echocardiography reading center in accordance with American Society of Echocardiography guidelines^25^, ^26^ by analysts blinded to clinical information. Mitral regurgitation was assessed based on mitral regurgitation jet area:left atrial area ratio. The regurgitant color Doppler signal was traced on systole in the apical 4‐ and parasternal 2‐chamber views, and the largest mitral regurgitation jet area:left atrial area ratio was used.^24^, ^27^ Mitral regurgitation severity category was increased by 1 if jet eccentricity was present. Aortic stenosis was defined using aortic valve peak velocity measured using the apical 5‐chamber view. Qualitative assessments of aortic regurgitation and mitral stenosis were performed by trained cardiologists based on color Doppler signal in the parasternal long‐ and short‐axis views and in the apical 5‐ and 3‐chamber views.

### Assessment of Covariates

Coronary heart disease was defined as having a history of prior adjudicated myocardial infarction or coronary intervention^28^ or a regional wall motion abnormality on echocardiography at Visit 5. Hypertension was based on self‐report, antihypertensive medication, or blood pressure ≥140/90 mm Hg at study Visit 5. Diabetes was defined based on self‐report, fasting glucose ≥126 mg/dL, or any glucose measurement ≥200 mg/dL. Prevalent HF was defined as hospitalization with an *International Classification of Diseases, Ninth Revision* (*ICD‐9*) code of 428 before 2005,^29^ with additional physician adjudication since 2005 as previously described.^30^ Atrial fibrillation diagnosis was based on ECG at Visits 1 through 5 and hospital discharge records as previously reported.^31^

### Statistical Analysis

Continuous variables are expressed as mean±SD for normally distributed data or median (25th, 75th percentiles) for nonnormally distributed data. Categorical variables are expressed as frequency and proportions. Clinical and demographic differences between robust, prefrail, and frail groups were evaluated with a trend test using linear and logistic regression for continuous and categorical variables, respectively.

To assess the association of prevalent frailty at Visit 5 with changes in cardiac structure and function from Visits 5 to 7, change in each echocardiographic measure was calculated as the Visit 7 measure minus the Visit 5 measure. Clinical characteristics and echocardiographic measures were described by frailty category at Visit 5. Echocardiographic data for each variable were normally or near‐normally distributed. Analyses were performed using multivariable linear regression models adjusted for age, sex, race, field center, blood pressure and heart rate at both echo visits, hypertension, diabetes, body mass index, estimated glomerular filtration rate, prevalent coronary heart disease, atrial fibrillation, history of myocardial infarction, and prevalent stroke assessed at Visit 5. Values from Visit 5, Visit 7, and change values were compared among Visit 5 frailty categories using multivariable linear regression. We then assessed the association of changes in frailty status between Visit 5 and Visit 7 with concomitant changes in echocardiographic measures. Participants were classified as robust at Visit 5 to robust at Visit 7, robust to prefrail, and robust to frail. Comparisons between groups stratified by Visit 5 frailty status were performed using multivariable linear regression, with stable frailty status as the reference group.

To assess the association of alterations in cardiac structure and function at Visit 5 with the progression in frailty status between Visits 5 and 6 among participants who were robust at Visit 5, we employed multivariable ordinal logistic regression models to assess the association between a 1‐SD increment in each Visit 5 echocardiographic measurement and the odds of progressing to prefrailty and frailty at V6 as an ordinal, 3‐level outcome (robust, prefrail, and frail). Two models were generated. The first adjusted demographic covariates (age, sex, race, and field center). The second additionally adjusted for blood pressure and heart rate, hypertension, diabetes, body mass index, estimated glomerular filtration rate, prevalent coronary heart disease, history of myocardial infarction, and prevalent stroke. To assess the robustness of our findings, we performed 3 sensitivity analyses: (1) excluding participants with moderate or severe valvulopathy, (2) excluding participants who self‐reported moderate or severe dyspnea based on the modified Medical Research Council questionnaire^32^, ^33^ and had an NT‐proBNP (N‐terminal pro‐B‐type natriuretic peptide) >450 pg/dL at Visit 5, and^3^ excluding participants with an H_2_FPEF score (Heavy, Hypertensive, Atrial Fibrillation, Pulmonary Hypertension, Elder, Filling Pressure) >6 at Visit 5.^34^, ^35^ An additional sensitivity analysis was performed to evaluate the associations of echocardiographic measures with incident frailty or prefrailty using separate logistic regression models.

A 2‐sided *P* value <0.05 was considered significant. Statistical analysis was performed using Stata software Version IC‐16.1 (Stata Corp LP, College Station, TX).

## Results

### Frailty Status and Longitudinal Changes in Cardiac Structure and Function

Among the 2574 participants with echocardiography at Visits 5 and 7 and free of HF at Visit 5, mean age was 74.5±4.4 at Visit 5 and 80.5±4.4 at Visit 7, 58% were female, and 22% reported Black race (Table 1). Frail participants at Visit 5 were more likely to die between Visits 5 and 7, and, among those alive at Visit 7, to not attend that visit. Baseline characteristics at Visit 5 of participants included in this analysis who died between Visit 5 and 7 and who were alive at Visit 7 but not included are shown in Table S1. At Visit 5, 1393 (54%) were robust, 1098 (43%) were prefrail, and 83 (3%) were frail. Frailty at Visit 5 was associated with older age, female sex, and higher prevalence of cardiovascular comorbidities (Table 1), and with greater left ventricular (LV) mass index, higher E/e', and larger LV end‐diastolic dimension, and left atrial volume index (LAVi) cross‐sectionally at Visit 5 (Table 2). Frailty at Visit 5 was also associated with greater left ventricular mass index, mean wall thickness, higher E/e', and larger LAVi at Visit 7. Worse frailty status at Visit 5 associated with greater increase in E/A ratio between Visits 5 and 7 but not with changes in other echocardiographic measures (Table 2).

**Table 1:** Baseline Characteristics of the Study Population for Changes in Echo Analysis at Visit 5 by Frailty Category, The Atherosclerosis Risk in Communities Study Cohort (2011–2013, N=2574)

	Overall	Robust	Prefrail	Frail	*P* for trend
	n=2574	n=1393	n=1098	n=83	
Age, mean±SD, y	74.0±4.4	73.4±4.1	74.7±4.6	75.3±4.8	<0.001
Male sex, n (%)	1093 (45.2%)	638 (45.8%)	430 (39.2%)	25 (30.1%)	<0.001
Black race, n (%)	571 (22.2%)	273 (19.6%)	271 (24.7%)	27 (32.5%)	<0.001
Hypertension, n (%)	1787 (69.8%)	917 (66.1%)	803 (73.7%)	67 (80.7%)	<0.001
Diabetes, n (%)	695 (27.0%)	307 (22.0%)	353 (32.1%)	35 (42.2%)	<0.001
Body mass index, mean±SD, kg/m^2^	28.8±5.2	28.3±4.8	29.2±5.5	31.5±7.2	<0.001
Estimated glomerular filtration rate by Chronic Kidney Disease Epidemiology Collaboration, mean±SD, mL/min per 1.73 m^2^	73.0±15.5	73.6±14.7	72.4±16.3	70.0±17.0	0.014
Coronary heart disease, n (%)	263 (10.4%)	123 (9.0%)	130 (12.1%)	10 (12.0%)	0.014
Atrial fibrillation, n (%)	97 (3.8%)	47 (3.4%)	46 (4.2%)	4 (4.8%)	0.5
Myocardial infarction, n (%)	211 (8.6%)	97 (7.3%)	105 (10.0%)	9 (11.2%)	0.015
Stroke, n (%)	60 (2.3%)	23 (1.7%)	34 (3.1%)	3 (3.6%)	0.014
Aortic or mitral valvular prosthesis	9 (0.3%)	4 (0.3%)	5 (0.5%)	0 (0%)	0.67
Moderate to severe aortic stenosis	10 (0.4%)	4 (0.3%)	5 (0.5%)	1 (1.2%)	0.24
Moderate–severe to severe mitral regurgitation	18 (0.7%)	9 (0.6%)	9 (0.6%)	0 (0%)	0.95
N‐terminal pro‐B‐type natriuretic peptide, median [IQR], pg/mL	99 [54–184]	88 [50–167]	113 [61–202]	147 [76–301]	<0.001

*P* values assess for trend for each variable across all categories. Data are shown as frequency and proportion or mean±SD or median (IQR [25th and 75th percentiles]). *P* values for trend were calculated using linear and logistic regression as appropriate. Right skewed variables were log transformed before testing for trend. IQR indicates interquartile range.

**Table 2:** Echo Measurements at Visit 5 and Visit 7 and Their Change, by Frailty Category at Visit 5

				*P* value for trend	
Category	Robust	Prefrail	Frail	Model 1	Model 2
	n=1366	n=1054	n=78		
LV mass index (g/m^2^)					
Visit 5	76.46±17.30	77.25±18.21	82.30±21.77	0.003	0.42
Visit 7	80.82±19.28	82.00±21.18	89.23±24.55	0.002	0.33
Delta	4.36±15.08	4.75±15.67	6.93±18.13	0.55	0.74

	n=1375	n=1065	n=81		
LV end‐diastolic dimension (cm)					
Visit 5	4.40±0.48	4.38±0.49	4.42±0.55	0.029	0.99
Visit 7	4.35±0.50	4.29±0.51	4.38±0.57	0.52	0.14
Delta	−0.05±0.35	−0.09±0.37	−0.04±0.28	0.06	0.07

	n=1380	n=1077	n=82		
Mean wall thickness (cm)					
Visit 5	0.97±0.13	0.98±0.13	1.00±0.12	0.06	0.18
Visit 7	1.02±0.15	1.03±0.15	1.06±0.14	0.003	0.72
Delta	0.05±0.12	0.06±0.12	0.06±0.12	0.11	0.09

	n=1313	n=10 006	n=77		
LV ejection fraction (%)					
Visit 5	65.66±5.62	65.68±5.82	65.37±7.10	0.43	0.57
Visit 7	63.25±6.91	63.44±7.22	62.15±7.71	0.75	0.88
Delta	−2.40±6.50	−2.24±6.57	−3.22±6.01	0.73	0.52

	n=1351	n=1043	n=78		
Left atrial volume index (mL/m^2^)					
Visit 5	24.74±7.32	25.18±7.56	27.39±8.24	0.006	0.045
Visit 7	27.28±9.01	28.15±9.26	30.99±10.54	0.002	0.033
Delta	2.53±6.93	2.96±6.97	3.60±7.26	0.25	0.51

	n=1374	n=1079	n=82		
e' lateral (cm/s)					
Visit 5	7.33±1.98	7.11±2.01	6.93±2.01	0.36	0.99
Visit 7	6.69±1.95	6.55±2.00	6.81±2.05	0.38	0.21
Delta	−0.63±2.08	−0.56±2.11	−0.12±2.17	0.09	0.24

	n=1366	n=1006	n=78		
E/e' lateral (ratio)					
Visit 5	9.46±3.17	10.08±3.68	10.50±3.54	0.003	0.34
Visit 7	11.80±4.65	12.63±4.99	12.43±4.30	0.031	0.59
Delta	2.34±3.93	2.54±4.22	1.94±3.80	0.95	0.88

	n=1297	n=999	n=76		
E/A ratio					
Visit 5	0.87±0.25	0.85±0.25	0.83±0.28	0.98	0.93
Visit 7	0.86±0.28	0.86±0.30	0.86±0.28	0.05	0.06
Delta	−0.01±0.27	0.01±0.30	0.03±0.31	0.04	0.045

*P* values provide significance for trend across frailty categories. Data are shown as mean±SE. Model 1 adjusts for demographics (age, sex, race, field center, heart rate, and blood pressure at echo Visits 5 and 7); Model 2 adjusts for demographics and heart rate and blood pressure at Visits 5 and 7. LV indicates left ventricular.

**Figure 2:**
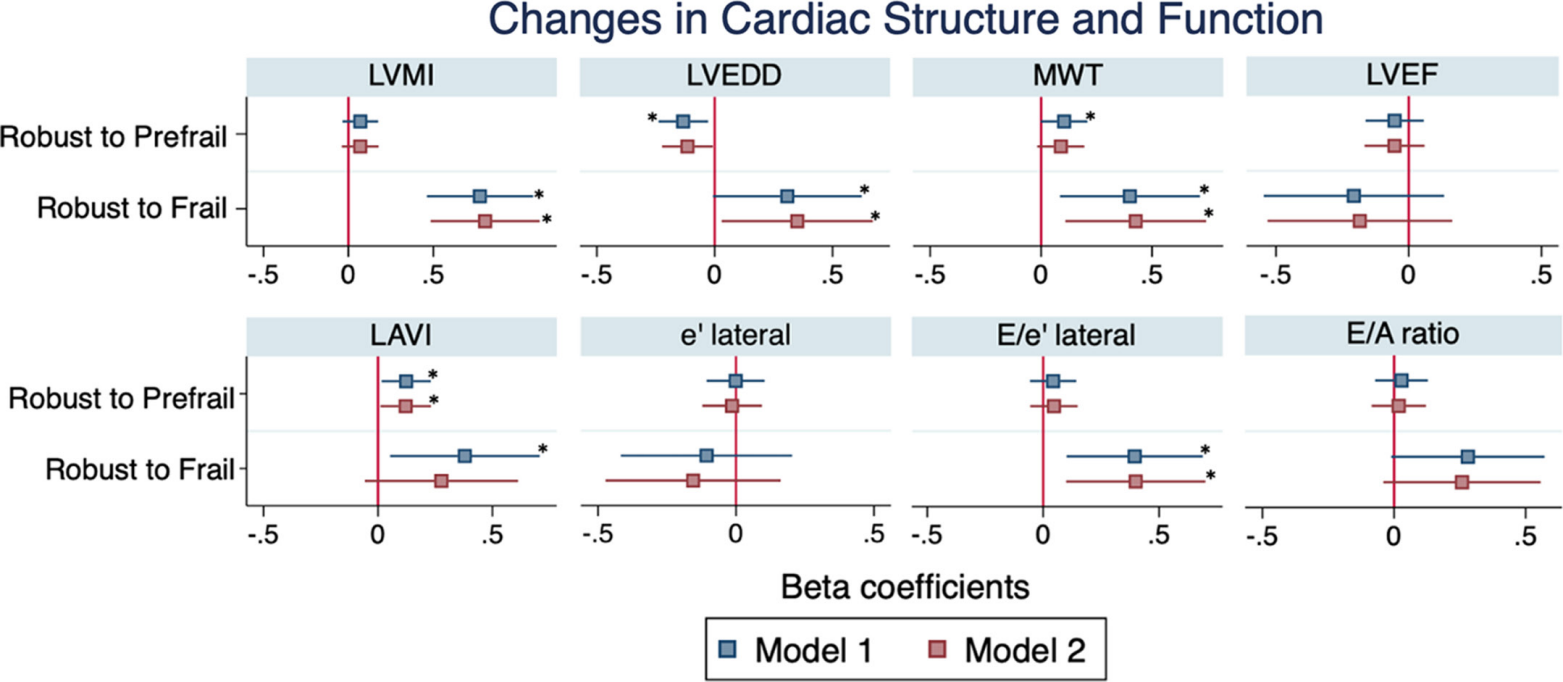
Associations of transitions in frailty status from Visit 5 to Visit 7 with concomitant changes in echocardiographic measures. Plot demonstrates model beta coefficients and 95% CI for echocardiographic measurements by changes in frailty status. Model 1 adjusts for demographics (age, sex, race, and field center), and heart rate and blood pressure at echo visits. Model 2 additionally adjusts comorbidities (body mass index, history of smoking, prevalent coronary heart disease, estimated glomerular filtration rate, diabetes, hypertension, history of stroke, and prevalent atrial fibrillation) at Visit 5. No change in frailty status between visits is taken as the reference value for each comparison (X line at 0). *Indicates significant at *P*<0.05. LAVI indicates left atrial volume index; LVEDD, left ventricular end diastolic dimension; LVEF, left ventricular mass index; LVMI, left ventricular mass index; and MWT, mean wall thickness.

Of 1393 participants who were robust at Visit 5, 683 (49%) and 43 (3%) progressed to prefrailty and frailty at Visit 7, respectively. Participants who transitioned from robust to frail demonstrated greater increases in LV mass index, end‐diastolic dimension, and wall thickness, and greater increases in measures of LV filling pressure (LAVi, E/e' ratio) compared with those who remained robust (Figure 2). No differences were observed in change in LV ejection fraction. Tables 3 and 4 additionally show the percentage of change between visits and comparisons between groups for robust at baseline and prefrail at baseline, respectively.

**Table 3:** Comparison of Echo Measurements at Visit 5 and Visit 7, Delta and Percent Change Across Changes in Frailty Status With Robust at Baseline

				*P* value for between groups			
Category	Robust‐robust	Robust‐prefrail	Robust‐frail	RR vs RP	RP vs RF	RR vs RF	*P* for trend
	n=657	n=668	n=41				
LV mass index (g/m^2^)							
Visit 5	76.07±0.64	76.66±0.63	77.10±2.56	0.59	0.68	0.86	0.50
Visit 7	79.44±0.71	81.09±0.71	93.10±2.88	0.14	0.001	0.001	0.001
Visit 7adj	79.69±0.56	80.85±0.56	92.91±2.27	0.15	0.001	0.001	0.001
Delta	3.37±0.59	4.43±0.59	16.01±2.40	0.23	0.001	0.001	0.001
% change	0.06±0.01	0.07±0.01	0.24±0.03	0.42	0.001	0.001	0.003

	n=661	n=673	n=41				
LV end‐diastolic dimension (cm)							
Visit 5	4.39±0.02	4.41±0.02	4.33±0.07	0.49	0.26	0.33	0.87
Visit 7	4.36±0.02	4.33±0.02	4.42±0.07	0.28	0.20	0.50	0.65
Visit 7adj	4.36±0.01	4.33±0.01	4.47±0.05	0.03	0.007	0.09	0.45
Delta	−0.03±0.01	−0.08±0.01	0.09±0.06	0.03	0.004	0.05	0.45
% change	−0.00±0.00	−0.01±0.00	0.02±0.01	0.024	0.003	0.049	0.41

	n=662	n=677	n=41				
Mean wall thickness (cm)							
Visit 5	0.97±0.00	0.97±0.00	1.00±0.02	0.4	0.08	0.43	0.96
Visit 7	1.01±0.01	1.02±0.01	1.08±0.02	0.47	0.002	0.002	0.032
Visit 7adj	1.01±0.00	1.02±0.00	1.07±0.02	0.1	0.05	0.002	0.013
Delta	0.04±0.00	0.05±0.00	0.09±0.02	0.10	0.049	0.009	0.012
% change	0.04±0.00	0.05±0.00	0.09±0.02	0.11	0.07	0.018	0.018

	n=643	n=634	n=36				
LV ejection fraction (%)							
Visit 5	65.60±0.22	65.68±0.22	66.27±0.93	0.87	0.42	0.83	0.63
Visit 7	63.41±0.27	63.14±0.27	62.75±1.14	0.44	0.9	0.49	0.42
Visit 7adj	63.44±0.24	63.12±0.24	62.59±1.02	0.34	0.57	0.4	0.25
Delta	−2.19±0.26	−2.54±0.26	−3.51±1.10	0.34	0.42	0.4	0.21
% change	−0.03±0.00	−0.04±0.00	−0.05±0.02	0.29	0.59	0.57	0.22

	n=654	n=658	n=39				
Left atrial volume index (mL/m^2^)							
Visit 5	24.41±0.27	25.10±0.27	25.19±1.12	0.10	0.87	0.51	0.08
Visit 7	26.49±0.34	28.01±0.34	29.20±1.41	0.002	0.5	0.041	0.001
Visit 7adj	26.80±0.27	27.71±0.27	28.90±1.10	0.025	0.46	0.037	0.004
Delta	2.08±0.27	2.90±0.27	4.01±1.13	0.024	0.50	0.07	0.015
% change	0.11±0.01	0.14±0.01	0.20±0.05	0.046	0.32	0.07	0.024

	n=658	n=674	n=42				
e' lateral (cm/s)							
Visit 5	7.28±0.08	7.35±0.08	7.27±0.30	0.50	0.84	0.77	0.66
Visit 7	6.68±0.07	6.71±0.07	6.47±0.30	0.67	0.47	0.36	0.97
Visit 7adj	6.70±0.07	6.69±0.07	6.44±0.27	0.86	0.48	0.4	0.82
Delta	−0.60±0.08	−0.63±0.08	−0.80±0.32	0.81	0.62	0.59	0.65
% change	−0.05±0.01	−0.05±0.01	−0.06±0.05	0.67	0.92	0.9	0.68

	n=657	n=669	n=42				
E/e' lateral (ratio)							
Visit 5	9.49±0.12	9.47±0.12	10.03±0.48	0.87	0.23	0.29	0.66
Visit 7	11.67±0.18	11.84±0.17	13.61±0.70	0.57	0.021	0.009	0.09
Visit 7adj	11.66±0.15	11.86±0.15	13.39±0.60	0.44	0.05	0.015	0.08
Delta	2.18±0.15	2.36±0.15	3.58±0.59	0.41	0.07	0.024	0.09
% change	0.28±0.02	0.30±0.02	0.36±0.06	0.48	0.42	0.25	0.27

	n=627	n=630	n=40				
E/A (ratio)							
Visit 5	0.88±0.01	0.86±0.01	0.91±0.04	0.05	0.09	0.85	0.2
Visit 7	0.86±0.01	0.84±0.01	0.96±0.04	0.18	0.006	0.07	0.91
Visit 7adj	0.86±0.01	0.85±0.01	0.95±0.04	0.68	0.029	0.048	0.55
Delta	−0.02±0.01	−0.02±0.01	0.05±0.04	0.7	0.12	0.08	0.3
% change	0.01±0.01	0.01±0.01	0.06±0.04	0.98	0.27	0.28	0.68

Visit 7 measurements adjusted for Visit 5 measurements are also provided. *P* values compare between each change in frailty group, and trend. All measurements are adjusted for demographics (age, sex, race, and field center), heart rate, and blood pressure at Visits 5 and 7, and comorbidities (body mass index, history of smoking, prevalent coronary heart disease, estimated glomerular filtration rate, diabetes, hypertension, history of stroke, prevalent atrial fibrillation). LV indicates left ventricular; RF, robust at Visit 5 and frail at Visit 7; RP, robust at Visit 5 and prefrail at Visit 7; and RR, robust at Visit 5 and robust at Visit 7.

**Table 4:** Comparison of Echo Measurements at Visit 5 and Visit 7, Delta and Percent Change Across Changes in Frailty Status With Prefrail at Baseline

				*P* value for between groups			
Category	Prefrail‐robust	Prefrail‐prefrail	Prefrail‐frail	PR vs PP	PP vs PF	PR vs PF	Trend
	n=229	n=722	n=103				
LV mass index (g/m^2^)							
Visit 5	75.30±1.15	77.75±0.64	78.52±1.81	0.049	0.73	0.25	0.06
Visit 7	79.19±1.32	82.41±0.74	84.48±2.08	0.027	0.34	0.07	0.015
Visit 7adj	80.68±1.00	82.05±0.56	83.54±1.58	0.22	0.33	0.16	0.11
Delta	3.89±1.04	4.66±0.58	5.96±1.64	0.52	0.40	0.29	0.30
% change	0.06±0.01	0.07±0.01	0.09±0.02	0.45	0.38	0.26	0.24

	n=230	n=730	n=105				
LV end‐diastolic dimension (cm)							
Visit 5	4.40±0.03	4.38±0.02	4.35±0.04	0.51	0.43	0.37	0.33
Visit 7	4.34±0.03	4.28±0.02	4.24±0.05	0.09	0.42	0.10	0.042
Visit 7adj	4.32±0.02	4.28±0.01	4.26±0.04	0.09	0.7	0.16	0.07
Delta	−0.07±0.02	−0.11±0.01	−0.11±0.04	0.17	0.96	0.31	0.18
% change	−0.01±0.01	−0.02±0.00	−0.02±0.01	0.20	0.94	0.34	0.24

	n=234	n=736	n=107				
Mean wall thickness (cm)							
Visit 5	0.96±0.01	0.98±0.00	0.99±0.01	0.015	0.33	0.039	0.008
Visit 7	1.01±0.01	1.04±0.01	1.06±0.01	0.002	0.24	0.008	0.001
Visit 7adj	1.02±0.01	1.04±0.00	1.05±0.01	0.035	0.43	0.06	0.018
Delta	0.05±0.01	0.06±0.00	0.06±0.01	0.27	0.72	0.41	0.23
% change	0.05±0.01	0.06±0.00	0.07±0.01	0.29	0.60	0.32	0.20

	n=224	n=686	n=96				
LV ejection fraction (%)							
Visit 5	65.51±0.38	65.75±0.21	64.85±0.61	0.62	0.16	0.60	0.66
Visit 7	63.12±0.46	63.74±0.26	61.56±0.73	0.27	0.004	0.30	0.43
Visit 7adj	63.17±0.41	63.67±0.23	61.99±0.65	0.32	0.012	0.37	0.51
Delta	−2.40±0.44	−2.00±0.25	−3.29±0.70	0.46	0.08	0.50	0.66
% change	−0.03±0.01	−0.03±0.00	−0.05±0.01	0.42	0.10	0.66	0.76

	n=228	n=715	n=100				
Left atrial volume index (mL/m^2^)							
Visit 5	24.46±0.48	25.40±0.27	25.11±0.76	0.08	0.60	0.51	0.22
Visit 7	26.89±0.59	28.36±0.33	29.23±0.94	0.021	0.45	0.048	0.016
Visit 7adj	27.44±0.46	28.17±0.26	29.27±0.73	0.12	0.14	0.05	0.035
Delta	2.43±0.47	2.95±0.26	4.12±0.75	0.27	0.12	0.09	0.07
% change	0.14±0.02	0.14±0.01	0.20±0.03	0.83	0.08	0.21	0.26

	n=234	n=741	n=104				
e' lateral (cm/s)							
Visit 5	7.11±0.13	7.11±0.07	7.15±0.21	0.92	0.96	0.92	0.93
Visit 7	6.54±0.13	6.58±0.07	6.64±0.21	0.81	0.82	0.69	0.68
Visit 7adj	6.54±0.12	6.58±0.07	6.63±0.19	0.82	0.82	0.69	0.67
Delta	−0.58±0.14	−0.53±0.08	−0.50±0.22	0.89	0.87	0.77	0.76
% change	−0.05±0.02	−0.04±0.01	−0.02±0.03	0.65	0.61	0.35	0.42

	n=232	n=735	n=103				
E/e' lateral (ratio)							
Visit 5	9.93±0.23	10.01±0.13	10.29±0.36	0.8	0.48	0.29	0.46
Visit 7	12.45±0.32	12.49±0.18	13.08±0.51	89.00	0.23	0.37	0.42
Visit 7adj	12.52±0.27	12.50±0.15	12.86±0.42	0.98	0.33	0.69	0.63
Delta	2.52±0.27	2.48±0.15	2.79±0.43	0.95	0.42	0.80	0.73
% change	0.31±0.03	0.31±0.02	0.33±0.05	0.84	0.59	0.82	0.68

	n=231	n=732	n=103				
E/A (ratio)							
Visit 5	0.86±0.02	0.84±0.01	0.93±0.03	0.33	0.002	0.11	0.24
Visit 7	0.88±0.02	0.85±0.01	0.91±0.03	0.22	0.13	0.84	0.88
Visit 7adj	0.88±0.02	0.86±0.01	0.87±0.03	0.35	0.84	0.66	0.49
Delta	0.03±0.02	0.01±0.01	−0.02±0.03	0.62	0.39	0.28	0.28
% change	0.10±0.03	0.05±0.02	0.03±0.05	0.14	0.74	0.42	0.14

Visit 7 measurements adjusted for Visit 5 measurements are also provided. *P* values compare between each change in frailty group, and trend. All measurements are adjusted for demographics (age, sex, race, and field center), heart rate, and blood pressure at Visits 5 and 7, and comorbidities (body mass index, history of smoking, prevalent coronary heart disease, estimated glomerular filtration rate, diabetes, hypertension, history of stroke, and prevalent atrial fibrillation). LV indicates left ventricular; PF, prefrail at Visit 5 and frail at Visit 7; PP, prefrail at Visit 5 and prefrail at Visit 7; and PR, prefrail at Visit 5 and robust at Visit 7.

Similar findings with respect to the association between frailty category and change in cardiac structure and function between Visits 5 and 7 were observed in sensitivity analyses excluding participants with moderate to severe dyspnea and NT‐proBNP >450 at Visit 5 (n=49; Table S2), or excluding participants with an H_2_FPEF score ≥6 at Visit 5 (n=63; Table S3). In addition, similar findings as our primary analysis with respect to the association between changes in frailty status and changes in cardiac structure and function between Visits 5 and 7 were observed in sensitivity analyses excluding participants with moderate to severe valvulopathy or prior valve replacement (n=35; Figure S1), participants with dyspnea and NT‐proBNP >450 (n=49; Figure S2), and participants with H_2_FPEF score >6 (n=63; Figure S3).

### Cardiac Structure and Function and Incident Frailty

**Figure 3:**
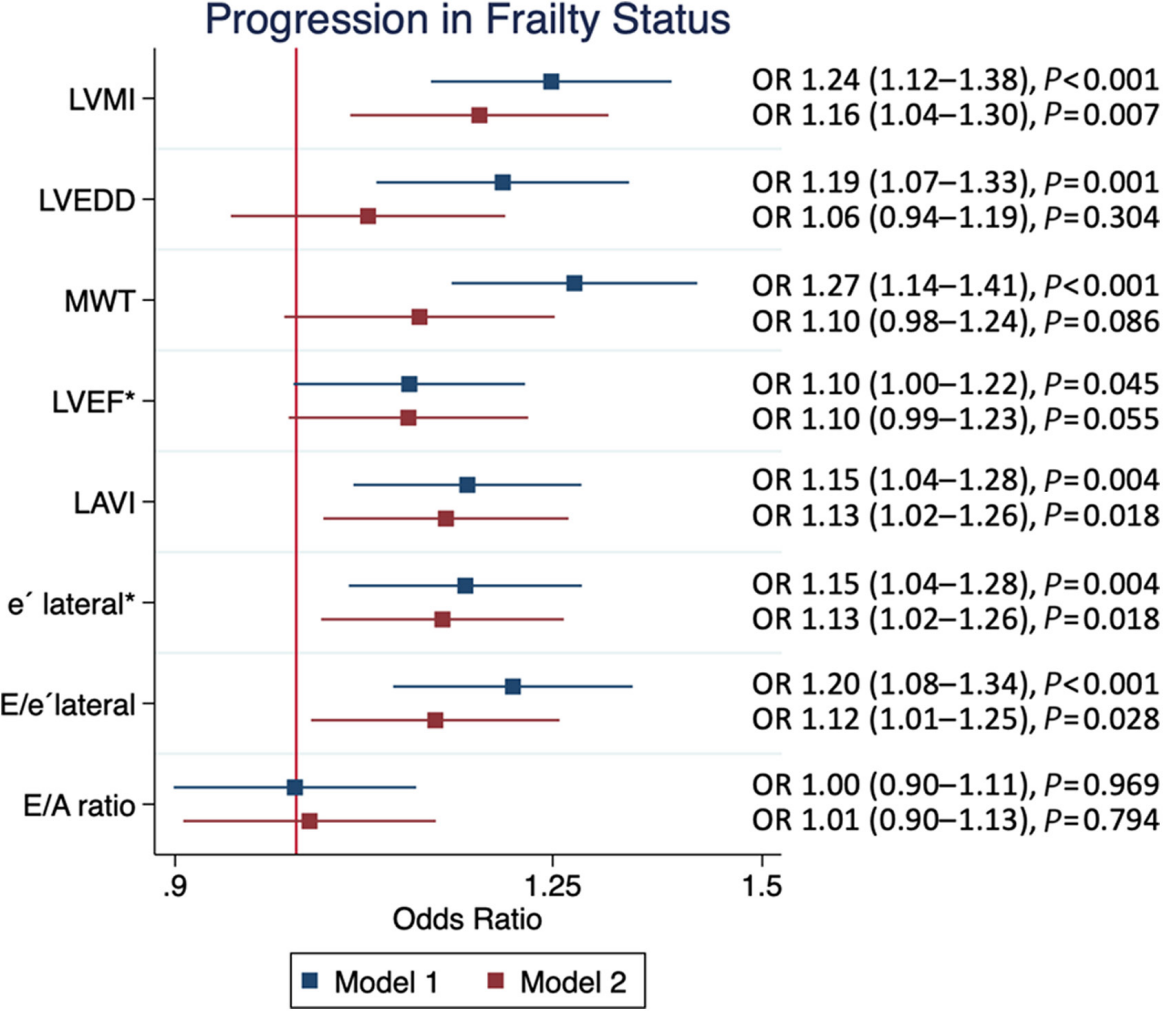
Association of measures of cardiac structure and function at Visit 5 with the progression in frailty status at Visit 6. Model 1 adjusts for demographics (age, sex, race, and field center), and echo visit heart rate and blood pressure. Model 2 additionally adjusts for comorbidities (body mass index, history of smoking, prevalent coronary heart disease, estimated glomerular filtration rate, diabetes, hypertension, prevalent stroke, and prevalent atrial fibrillation) at Visit 5. *Indicates odds associated with lower values. LAVI indicates left atrial volume index; LVEDD, left ventricular end diastolic dimension; LVMI, left ventricular mass index; LVEF, left ventricular mass index; MWT, mean wall thickness; and OR, odds ratio.

Among 2549 participants free of HF who were robust and had an echocardiographic assessment at Visit 5, 213 died between Visit 5 and Visit 6, and frailty at Visit 6 was not assessed in 688 participants. Among the 1648 participants who were included in this analysis, 754 (45%) developed prefrailty at Visit 6, and 49 (3%) developed frailty. Baseline characteristics of this population at Visit 5 and comparisons by their frailty status at Visit 6 are shown in Table S4. Baseline characteristics at Visit 5 of participants included in this analysis who died between Visit 5 and 6 and who were alive at Visit 6 but not included are shown in Table S5. In models adjusted for demographics and clinical comorbidities, greater left ventricular mass index, and tissue Doppler imaging e', and higher LAVi and E/e' ratio were associated with higher odds of progressing in frailty status (Figure 3). These associations appeared driven by associations with incident frailty, with smaller magnitudes of effect for associations with incident prefrailty (Figure S4A and S4B). Echocardiographic measures demonstrated differential associations with progression in component frailty measures (Figure S5). Higher LVMI and mean wall thickness and lower tissue Doppler imaging e' were associated with development of low gait speed, greater LAVi was associated with development of low grip strength, and measures of higher LV filling pressure (E/e', E/A ratio) were associated with development of exhaustion. Similar findings were observed in sensitivity analyses excluding participants with greater than moderate valvular disease or prior valve replacement (n=22; Figure S6), excluding participants with moderate to severe dyspnea and NT‐proBNP >450 (n=20; Figure S7) and participants with H_2_FPEF score ≥6 (n=42; Figure S8).

## Discussion

Aging is an important risk factor for frailty, HF, and alterations in cardiac structure and function underlying HF. By leveraging longitudinal cardiovascular and frailty phenotyping in a community‐based cohort of older adults, we describe the following novel findings on the intersection of frailty and cardiac function among robust older adults. First, the presence of a greater LV mass index, worse diastolic function, and higher E/e' ratio is associated with a greater likelihood of progressing to a worse frailty status over a ≈5‐year follow‐up. Second, over a ≈ 6‐year follow‐up, frailty was associated with greater LV mass index and measures of LV filling pressure (E/e' ratio, LAVi) at baseline and follow‐up, and the transition from robust to frail during this time frame was characterized by greater increases in LV size, wall thickness, and mass index and by greater increases in measures of LV filling pressure, including LAVi and E/e' ratio. Transition from robust to prefrail was associated with greater increases in wall thickness and LAVi but of lesser magnitude. Finally, these associations persisted in models accounting for traditional cardiovascular risk factors and in 3 different sensitivity analyses excluding participants with significant left heart valvular disease or participants at risk of HF with prior hospitalization. Together, these findings suggest bidirectional associations between frailty and subclinical LV remodeling and diastolic dysfunction among older adults. Importantly, frailty status and cardiac structure and function are modifiable. Our findings raise the hypothesis that interventions to modify one may exert beneficial effects on the other.

The interrelationship between frailty and HF is well described.^36^, ^37^ Among patients with HF, frailty is common and is associated with higher risk of adverse outcomes compared with those without frailty.^12^, ^38^, ^39^ Moreover, prevalent HF is associated with a heightened risk of incident frailty in later life,^40^ as are subclinical measures of arteriosclerosis based on carotid intima‐media thickness.^41^ Conversely, among older adults free of HF, prevalent frailty is associated with greater risk of incident HF, independent of traditional cardiovascular risk factors.^13^, ^42^, ^43^ Our findings are novel in that we show a longitudinal association between subclinical cardiac dysfunction and clinical frailty phenotype. The mechanisms underlying these associations are unclear. Both frailty and HF are systemic disorders, and impairments in several organ functions are likely shared. Alterations in cardiac structure and function underlie the development of HF and are robust risk factors for HF development. In this context, among older adults free of HF, frailty is also cross‐sectionally associated with greater LV remodeling and dysfunction—specifically greater LV hypertrophy, diastolic dysfunction, and subtle impairments in LV deformation by strain imaging.^16^, ^44^, ^45^, ^46^ However, these findings are limited by potential reverse‐causation, and there are few data to date regarding the temporal sequence between development of the frailty phenotype and of alterations in cardiac structure and function.

Recent data from the Medical Research Council British National Survey of Health and Development indicate an association between severity of frailty ascertained by the frailty index in midlife and greater LV mass index and lower LV ejection fraction in later‐life.^47^ Our findings now demonstrate that greater increases in LV mass index and metrics of LV filling pressure accompany transitions from robust to frail and suggest a potential mechanism underlying the association of frailty with HF incidence. The persistence of these associations after accounting for traditional cardiovascular risk factors suggests that these findings may not be simply the result of shared risk factors for frailty and LV diastolic dysfunction in late life. Notably, among robust older adults, we also found that greater LV mass index and worse diastolic measures were associated with a higher likelihood of developing frailty, consistent with at least 1 prior study suggesting an association of subclinical cardiovascular disease in the form of carotid atherosclerosis with the development of frailty.^41^ These findings extend existing cross‐sectional studies to suggest bidirectional associations between LV diastolic dysfunction and frailty in late life, whereby the presence of one promotes development of the other.

The mechanisms linking frailty and cardiac alterations are unclear, although they are known to share common risk factors.^11^, ^48^ Alternatively, frailty and HF have also been associated with systemic inflammation,^18^ and comorbidity‐driven inflammation may be an important common pathobiology.^17^, ^19^, ^49^ Given the systemic, multiorgan nature of both frailty and HF, future studies leveraging high throughput‐omics data (proteomics, metabolomics) hold promise in more deeply interrogating the potential role of inflammation and in discovering novel underlying molecular pathways. Importantly, frailty is modifiable^50^ and potentially reversible.^3^ Transitions from frailty to prefrailty or robust are well described in observational studies^51^ and may be promoted by interventions mainly related to physical exercise.^52^, ^53^ In fact, exercise interventions appear efficacious for relevant clinical outcomes in HF and may promote improvements in LV function in HF with reduced ejection fraction.^54^ Whether interventions aimed at improving frail individuals to nonfrail status will also prove effective at preventing the development of HF—and the underlying alterations in cardiac structure and function—is unknown.

This study has several limitations. This is an analysis of an observational cohort of community‐dwelling participants, and therefore we can only identify associations but cannot draw conclusions regarding causality. Attrition between V5 and V7 due to death or nonattendance was differential with respect to frailty categories, and participants more likely to develop frailty were less likely to have follow‐up data. The differential attrition therefore limited the statistical power and likely biased our results toward the null. Our analytic approach did not account for this source of bias, which likely makes our estimates more conservative. In addition, a relatively small number of participants who progressed from robust to frail between Visits 5 and 7 (n=43, 3%) limited the power of our analysis and increased the likelihood of type II error. The high incidence of mortality and study attrition in very late life highlights the importance of future studies evaluating intermediate frailty phenotypes. Frailty status was determined using one particular metric,^2^ and whether these results would be consistent using other metrics of frailty remains unknown. Nonetheless, the Fried frailty phenotype has been previously operationalized and validated in our cohort^22^ and is commonly employed in other community‐based cohorts.^16^, ^23^, ^55^ Unmeasured confounding by shared cardiovascular risk factors cannot be excluded as a potential explanation for the observed associations between frailty and cardiac structure and function in this observational analysis. Echocardiography was not available in ARIC before study Visit 5, limiting our ability to assess the associations of frailty development with alterations in cardiac structure and function over longer duration. The relative small number of participants who demonstrated regression in frailty category between Visit 5 and Visit 7 (ie, from frail to prefrail [n=54] or to robust [n=6]) limited our ability to assess for changes in cardiac structure and function associated with improvements in frailty status.

## Conclusions

Among robust older adults in the community who are HF free, a bidirectional relationship exists between the development of frailty and subclinical LV remodeling and diastolic dysfunction. As both frailty status and cardiac structure and function are modifiable, future studies should evaluate whether interventions to modify one may exert beneficial effects on the other.

## Sources of Funding

The Atherosclerosis Risk in Communities study has been funded in whole or in part with federal funds from the National Heart, Lung, and Blood Institute, National Institutes of Health, Department of Health and Human Services, under contract nos. (HHSN268201700001I, HHSN268201700002I, HHSN268201700003I, HHSN268201700005I, HHSN268201700004I). This work was also supported by NIH/NHLBI grants R01HL135008 (A.M.S.), R01HL143224 (A.M.S.), R01HL150342 (A.M.S.), R01HL148218 (A.M.S.), and K24HL152008 (A.M.S.).

## Disclosures

Dr Shah reports consulting fees from Philips Ultrasound and research funds from Novartis through Brigham and Women's Hospital. The remaining authors have no disclosures to report.

## Supporting information

Tables S1‐S5
Figures S1‐S8

## References

[1] Sander M , Oxlund B , Jespersen A , Krasnik A , Mortensen EL , Westendorp RGJ , Rasmussen LJ . The challenges of human population ageing. Age Ageing. 2015;44:185–187. doi: 10.1093/ageing/afu189 25452294 PMC4339729

[2] Fried LP , Tangen CM , Walston J , Newman AB , Hirsch C , Gottdiener J , Seeman T , Tracy R , Kop WJ , Burke G , et al. Frailty in older adults: evidence for a phenotype. J Gerontol A Biol Sci Med Sci. 2001;56:M146–M157. doi: 10.1093/gerona/56.3.M146 11253156

[3] Gill TM , Gahbauer EA , Allore HG , Han L . Transitions between frailty states among community‐living older persons. Arch Intern Med. 2006;166:6. doi: 10.1001/archinte.166.4.418 16505261

[4] Siriwardhana DD , Hardoon S , Rait G , Weerasinghe MC , Walters KR . Prevalence of frailty and prefrailty among community‐dwelling older adults in low‐income and middle‐income countries: a systematic review and meta‐analysis. BMJ Open. 2018;8:e018195. doi: 10.1136/bmjopen-2017-018195 PMC5855322 29496895

[5] Shah AM , Claggett B , Kitzman D , Biering‐Sørensen T , Jensen JS , Cheng S , Matsushita K , Konety S , Folsom AR , Mosley TH , et al. Contemporary assessment of left ventricular diastolic function in older adults: the Atherosclerosis Risk in Communities study. Circulation. 2017;135:426–439. doi: 10.1161/CIRCULATIONAHA.116.024825 27927714 PMC5285443

[6] Kitzman DW , Sheikh KH , Beere PA , Philips JL , Higginbotham MB . Age‐related alterations of doppler left ventricular filling indexes in normal subjects are independent of left ventricular mass, heart rate, contractility and loading conditions. J Am Coll Cardiol. 1991;18:1243–1250. doi: 10.1016/0735-1097(91)90542-H 1918701

[7] Halley CM , Houghtaling PL , Khalil MK , Thomas JD , Jaber WA . Mortality rate in patients with diastolic dysfunction and normal systolic function. Arch Intern Med. 2011;171:1082–1087. 21709107 10.1001/archinternmed.2011.244

[8] Reimer Jensen AM , Zierath R , Claggett B , Skali H , Solomon SD , Matsushita K , Konety S , Butler K , Kitzman DW , Biering‐Sørensen T , et al. Association of left ventricular systolic function with incident heart failure in late life. JAMA Cardiol. 2021;6:509. doi: 10.1001/jamacardio.2021.0131 33729428 PMC7970394

[9] Aurigemma GP , Gottdiener JS , Arnold AM , Chinali M , Hill JC , Kitzman D . Left atrial volume and geometry in healthy aging: the cardiovascular health study. Circ. Cardiovascular Imaging. 2009;2:282–289. doi: 10.1161/CIRCIMAGING.108.826602 19808608 PMC4156514

[10] Sze S , Pellicori P , Zhang J , Weston J , Clark AL . Identification of frailty in chronic heart failure. JACC: Heart Fail. 2019;7:291–302. 30738977 10.1016/j.jchf.2018.11.017

[11] Uchmanowicz I , Nessler J , Gobbens R , Gackowski A , Kurpas D , Straburzynska‐Migaj E , Kałuzna‐Oleksy M , Jankowska EA . Coexisting frailty with heart failure. Front Physiol. 2019;10:791. doi: 10.3389/fphys.2019.00791 31333480 PMC6616269

[12] Cacciatore F , Abete P , Mazzella F , Viati L , Della Morte D , D'Ambrosio D , Gargiulo G , Testa G , Santis D , Galizia G , et al. Frailty predicts long‐term mortality in elderly subjects with chronic heart failure. Eur J Clin Investig. 2005;35:723–730. doi: 10.1111/j.1365-2362.2005.01572.x 16313247

[13] McKechnie DG , Papacosta AO , Lennon LT , Ramsay SE , Whincup PH , Wannamethee SG . Frailty and incident heart failure in older men: the British regional heart study. Open Heart. 2021;8:e001571. doi: 10.1136/openhrt-2021-001571 34088788 PMC8183233

[14] Veronese N , Cereda E , Stubbs B , Solmi M , Luchini C , Manzato E , Sergi G , Manu P , Harris T , Fontana L , et al. Risk of cardiovascular disease morbidity and mortality in frail and pre‐frail older adults: results from a meta‐analysis and exploratory meta‐regression analysis. Ageing Res Rev. 2017;35:63–73. doi: 10.1016/j.arr.2017.01.003 28143778 PMC6047747

[15] Salminen M , Viljanen A , Eloranta S , Viikari P , Wuorela M , Vahlberg T , Isoaho R , Kivelä SL , Korhonen P , Irjala K , et al. Frailty and mortality: an 18‐year follow‐up study among Finnish community‐dwelling older people. Aging Clin Exp Res. 2020;32:2013–2019. doi: 10.1007/s40520-019-01383-4 31654244 PMC7532963

[16] Newman AB , Gottdiener JS , McBurnie MA , Hirsch CH , Kop WJ , Tracy R , Walston JD , Fried LP . Associations of subclinical cardiovascular disease with frailty. J Gerontol Ser A Biol Med Sci. 2001;56:M158–M166. doi: 10.1093/gerona/56.3.M158 11253157

[17] Franceschi C , Garagnani P , Parini P , Giuliani C , Santoro A . Inflammaging: a new immune–metabolic viewpoint for age‐related diseases. Nat Rev Endocrinol. 2018;14:576–590. doi: 10.1038/s41574-018-0059-4 30046148

[18] Bellumkonda L , Tyrrell D , Hummel SL , Goldstein DR . Pathophysiology of heart failure and frailty: a common inflammatory origin? Aging Cell. 2017;16:444–450. doi: 10.1111/acel.12581 28266167 PMC5418206

[19] Cesari M , Penninx BWJH , Pahor M , Lauretani F , Corsi AM , Williams GR , Guralnik JM , Ferrucci L . Inflammatory markers and physical performance in older persons: the InCHIANTI study. J Gerontol Ser A Biol Med Sci. 2004;59:M242–M248. doi: 10.1093/gerona/59.3.M242 15031308

[20] The atherosclerosis risk in communities (ARIC) study: design and objectives. Am J Epidemiol. 1989;129:687–702. doi: 10.1093/oxfordjournals.aje.a115184 2646917

[21] Wright JD , Folsom AR , Coresh J , Sharrett AR , Couper D , Wagenknecht LE , Mosley TH , Ballantyne CM , Boerwinkle EA , Rosamond WD , et al. The ARIC (Atherosclerosis Risk In Communities) study. J Am Coll Cardiol. 2021;77:2939–2959. doi: 10.1016/j.jacc.2021.04.035 34112321 PMC8667593

[22] Kucharska‐Newton AM , Palta P , Burgard S , Griswold ME , Lund JL , Capistrant BD , Kritchevsky SB , Bandeen‐Roche K , Windham BG . Operationalizing frailty in the Atherosclerosis Risk in Communities study cohort. J Gerontol A Biol Sci Med Sci. 2016;72:glw144–glw388. doi: 10.1093/gerona/glw144 PMC6075198 27470301

[23] Bandeen‐Roche K , Xue QL , Ferrucci L , Walston J , Guralnik JM , Chaves P , Zeger SL , Fried LP . Phenotype of frailty: characterization in the women's health and aging studies. J Gerontol Ser A Biol Med Sci. 2006;61:262–266. doi: 10.1093/gerona/61.3.262 16567375

[24] Shah AM , Cheng S , Skali H , Wu J , Mangion JR , Kitzman D , Matsushita K , Konety S , Butler KR , Fox ER , et al. Rationale and design of a multicenter echocardiographic study to assess the relationship between cardiac structure and function and heart failure risk in a biracial cohort of community‐dwelling elderly persons: the Atherosclerosis Risk in Communities study. Circ Cardiovasc Imaging. 2014;7:173–181. doi: 10.1161/CIRCIMAGING.113.000736 24214885 PMC4467788

[25] Lang RM , Badano LP , Mor‐Avi V , Afilalo J , Armstrong A , Ernande L , Flachskampf FA , Foster E , Goldstein SA , Kuznetsova T , et al. Recommendations for cardiac chamber quantification by echocardiography in adults: an update from the American Society of Echocardiography and the European Association of Cardiovascular Imaging. J Am Soc Echocardiogr. 2015;28:1–39.e14. doi: 10.1016/j.echo.2014.10.003 25559473

[26] Nagueh SF , Smiseth OA , Appleton CP , Byrd BF , Dokainish H , Edvardsen T , Flachskampf FA , Gillebert TC , Klein AL , Lancellotti P , et al. Recommendations for the evaluation of left ventricular diastolic function by echocardiography: an update from the American Society of Echocardiography and the European Association of Cardiovascular Imaging. J Am Soc Echocardiogr. 2016;29:277–314. doi: 10.1016/j.echo.2016.01.011 27037982

[27] Zoghbi W . Recommendations for evaluation of the severity of native valvular regurgitation with two‐dimensional and doppler echocardiography. J Am Soc Echocardiogr. 2003;16:777–802. doi: 10.1016/S0894-7317(03)00335-3 12835667

[28] White AD , Folsom AR , Chambless LE , Sharret AR , Yang K , Conwill D , Higgins M , Williams OD , Tyroler HA . The ARIC investigators. Community surveillance of coronary heart disease in the Atherosclerosis Risk in Communities (ARIC) study: methods and initial two years' experience. J Clin Epidemiol. 1996;49:223–233. doi: 10.1016/0895-4356(95)00041-0 8606324

[29] Loehr LR , Rosamond WD , Chang PP , Folsom AR , Chambless LE . Heart failure incidence and survival (from the Atherosclerosis Risk in Communities Study). Am J Cardiol. 2008;101:1016–1022. doi: 10.1016/j.amjcard.2007.11.061 18359324

[30] Rosamond WD , Chang PP , Baggett C , Johnson A , Bertoni AG , Shahar E , Deswal A , Heiss G , Chambless LE . Classification of heart failure in the Atherosclerosis Risk In Communities (ARIC) study: a comparison of diagnostic criteria. Circ Heart Fail. 2012;5:152–159. 22271752 10.1161/CIRCHEARTFAILURE.111.963199 PMC3326579

[31] Alonso A , Agarwal SK , Soliman EZ , Ambrose M , Chamberlain AM , Prineas RJ , Folsom AR . Incidence of atrial fibrillation in whites and African‐Americans: the Atherosclerosis Risk in Communities (ARIC) study. Am Heart J. 2009;158:111–117. doi: 10.1016/j.ahj.2009.05.010 19540400 PMC2720573

[32] Fletcher CM , Elmes PC , Fairbairn AS , Wood CH . Significance of respiratory symptoms and the diagnosis of chronic bronchitis in a working population. BMJ. 1959;2:257–266. doi: 10.1136/bmj.2.5147.257 13823475 PMC1990153

[33] Ramalho SHR , Santos M , Claggett B , Matsushita K , Kitzman DW , Loehr L , Solomon SD , Skali H , Shah AM . Association of undifferentiated dyspnea in late life with cardiovascular and noncardiovascular dysfunction: a cross‐sectional analysis from the ARIC study. JAMA Netw Open. 2019;2:e195321. doi: 10.1001/jamanetworkopen.2019.5321 31199443 PMC6575149

[34] Reddy YNV , Carter RE , Obokata M , Redfield MM , Borlaug BA . A simple, evidence‐based approach to help guide diagnosis of heart failure with preserved ejection fraction. Circulation. 2018;138:861–870. doi: 10.1161/CIRCULATIONAHA.118.034646 29792299 PMC6202181

[35] Selvaraj S , Myhre PL , Vaduganathan M , Claggett BL , Matsushita K , Kitzman DW , Borlaug BA , Shah AM , Solomon SD . Application of diagnostic algorithms for heart failure with preserved ejection fraction to the community. JACC Heart Failure. 2020;8:640–653. doi: 10.1016/j.jchf.2020.03.013 32535127 PMC8030634

[36] Bekfani T , Pellicori P , Morris DA , Ebner N , Valentova M , Steinbeck L , Wachter R , Elsner S , Sliziuk V , Schefold JC , et al. Sarcopenia in patients with heart failure with preserved ejection fraction: impact on muscle strength, exercise capacity and quality of life. Int J Cardiol. 2016;222:41–46. doi: 10.1016/j.ijcard.2016.07.135 27454614

[37] Hegde SM , Gonçalves A , Claggett B , Evenson KR , Cheng S , Shah AM , Folsom AR , Solomon SD . Cardiac structure and function and leisure‐time physical activity in the elderly: the Atherosclerosis Risk in Communities study. Eur Heart J. 2016;37:2544–2551. doi: 10.1093/eurheartj/ehw121 27071820 PMC5008418

[38] Haehling S , Garfias Macedo T , Valentova M , Anker MS , Ebner N , Bekfani T , Haarmann H , Schefold JC , Lainscak M , Cleland JGF , et al. Muscle wasting as an independent predictor of survival in patients with chronic heart failure. J Cachexia Sarcopenia Muscle. 2020;11:1242–1249. 32767518 10.1002/jcsm.12603 PMC7567155

[39] Goldfarb M , Sheppard R , Afilalo J . Prognostic and therapeutic implications of frailty in older adults with heart failure. Curr Cardiol Rep. 2015;17:92. doi: 10.1007/s11886-015-0651-3 26346250

[40] Wang WJ , Lessard D , Saczynski J , Goldberg RJ , Go AS , Paul T , Gracia E , McManus DD . Incident frailty and cognitive impairment by heart failure status in older patients with atrial fibrillation: the SAGE‐AF study. J Geriatr Cardiol. 2020;17:653–658. doi: 10.11909/j.issn.1671-5411.2020.11.007 33343643 PMC7729184

[41] McKechnie DGJ , Papacosta AO , Lennon LT , Ellins EA , Halcox JPJ , Ramsay SE , Whincup PH , Wannamethee SG . Subclinical cardiovascular disease and risk of incident frailty: the British regional heart study. Exp Gerontol. 2021;154:111522. doi: 10.1016/j.exger.2021.111522 34428478

[42] Segar MW , Singh S , Goyal P , Hummel SL , Maurer MS , Forman DE , Butler J , Pandey A . Prefrailty, impairment in physical function, and risk of incident heart failure among older adults. J Am Geriatr Soc. 2021;69:2486–2497. 34050919 10.1111/jgs.17218 PMC10535362

[43] Khan H , Kalogeropoulos AP , Georgiopoulou VV , Newman AB , Harris TB , Rodondi N , Bauer DC , Kritchevsky SB , Butler J . Frailty and risk for heart failure in older adults: the health, aging, and body composition study. Am Heart J. 2013;166:887–894. doi: 10.1016/j.ahj.2013.07.032 24176445 PMC3844525

[44] Nadruz W , Kitzman D , Windham BG , Kucharska‐Newton A , Butler K , Palta P , Griswold ME , Wagenknecht LE , Heiss G , Solomon SD , et al. Cardiovascular dysfunction and frailty among older adults in the community: the ARIC study. Geron. 2016;72:glw199–glw964. doi: 10.1093/gerona/glw199 PMC5458397 27733425

[45] Gharacholou SM , Tashiro T , Cha SS , Scott CG , Takahashi PY , Pellikka PA . Echocardiographic indices associated with frailty in adults ≥65 years. Am J Cardiol. 2015;116:1591–1595. doi: 10.1016/j.amjcard.2015.08.023 26394832

[46] Arbab‐Zadeh A , Dijk E , Prasad A , Fu Q , Torres P , Zhang R , Thomas JD , Palmer D , Levine BD . Effect of aging and physical activity on left ventricular compliance. Circulation. 2004;110:1799–1805. doi: 10.1161/01.CIR.0000142863.71285.74 15364801

[47] Topriceanu CC , Moon JC , Hardy R , Chaturvedi N , Hughes AD , Captur G . Longitudinal birth cohort study finds that life‐course frailty associates with later‐life heart size and function. Sci Rep. 2021;11:6272. doi: 10.1038/s41598-021-85435-8 33737563 PMC7973558

[48] Marengoni A , Zucchelli A , Vetrano DL , Aloisi G , Brandi V , Ciutan M , Panait CL , Bernabei R , Onder G , Palmer K . Heart failure, frailty, and pre‐frailty: a systematic review and meta‐analysis of observational studies. Int J Cardiol. 2020;316:161–171. doi: 10.1016/j.ijcard.2020.04.043 32320778

[49] Al Saedi A , Feehan J , Phu S , Duque G . Current and emerging biomarkers of frailty in the elderly. Cia. 2019;14:389–398. doi: 10.2147/CIA.S168687 PMC6388773 30863033

[50] Kojima G . Frailty as a predictor of fractures among community‐dwelling older people: a systematic review and meta‐analysis. Bone. 2016;90:116–122. doi: 10.1016/j.bone.2016.06.009 27321894

[51] Ofori‐Asenso R , Chin KL , Mazidi M , Zomer E , Ilomaki J , Zullo AR , Gasevic D , Ademi Z , Korhonen MJ , LoGiudice D , et al. Global incidence of frailty and prefrailty among community‐dwelling older adults: a systematic review and meta‐analysis. JAMA Netw Open. 2019;2:e198398. doi: 10.1001/jamanetworkopen.2019.8398 31373653 PMC6681553

[52] Ahmad NS , Hairi NN , Said MA , Kamaruzzaman SB , Choo WY , Hairi F , Othman S , Ismail N , Peramalah D , Kandiben S , et al. Prevalence, transitions and factors predicting transition between frailty states among rural community‐dwelling older adults in Malaysia. PLoS One. 2018;13:e0206445. doi: 10.1371/journal.pone.0206445 30395649 PMC6218037

[53] Johnson RL , Frank RD , Abdel MP , Habermann EB , Chamberlain AM , Mantilla CB . Frailty transitions one year after total joint arthroplasty: a cohort study. J Arthroplast. 2022;37:10–18.e2. doi: 10.1016/j.arth.2021.08.022 34531097

[54] Haykowsky MJ , Liang Y , Pechter D , Jones LW , McAlister FA , Clark AM . A meta‐analysis of the effect of exercise training on left ventricular remodeling in heart failure patients. J Am Coll Cardiol. 2007;49:2329–2336. doi: 10.1016/j.jacc.2007.02.055 17572248

[55] Blodgett J , Theou O , Kirkland S , Andreou P , Rockwood K . Frailty in NHANES: comparing the frailty index and phenotype. Arch Gerontol Geriatr. 2015;60:464–470. doi: 10.1016/j.archger.2015.01.016 25697060

