# Supplementary material for: Bidirectional Association Between Frailty and Cardiac Structure and Function: The Atherosclerosis Risk in Communities Study
Source: J Am Heart Assoc. 2023 Jul 31;12(15):e029458. doi: 10.1161/JAHA.122.029458 (PMC10492980; doi:10.1161/JAHA.122.029458)

SUPPLEMENTAL MATERIAL

Table S1. Visit 5 characteristics of the population included for analyses 1 and 2. P-values compare participants included in the analysis to (1) participants alive but excluded from the analysis, and (2) participants who died between Visit 5 and Visit 7.

V5 Variable	Included in analysis	Alive at V7 but Excluded	p-value	Died Between V5 and V7	p-value
	n= 2574	n= 2,527	(Included vs Alive at Visit 7 but excluded)	n= 1437	(Included vs Died between V5 and V7)
Age	74 ± 4	76 ± 5	< 0.001	79 ± 6	< 0.001
Male	1093 (43%)	908 (36%)	< 0.001	692 (49%)	< 0.001
Black	571 (22%)	672 (27%)	< 0.001	300 (20%)	0.34
Hypertension	1787 (70%)	1926 (77%)	< 0.001	1112 (80%)	< 0.001
Diabetes	695 (27%)	849 (34%)	< 0.001	603 (42%)	< 0.001
BMI	29 ± 5	29 ± 6	0.043	28 ± 6	< 0.001
eGFR	73 ± 16	69 ± 17	< 0.001	62 ± 20	< 0.001
CHD	263 (10%)	363 (15%)	< 0.001	355 (25%)	< 0.001
MI	211 (9%)	301 (13%)	< 0.001	279 (21%)	< 0.001
Stroke	60 (2%)	116 (5%)	< 0.001	98 (7%)	< 0.001

Table S2: Echo measurements at Visit 5 and Visit 7 and their change, by frailty category at Visit 5 excluding participants with moderate to severe dyspnea and NT-pro BNP ≥450pg/dl.

	Category	Robust	Pre-frail	Frail	p-value for trend Model 1	p-value for trend Model 2
**LV mass index (g/m²)**	n=	1353	1026	71		
	Visit 5	76.40 ± 17.34	76.82 ± 17.48	80.84 ± 20.51	0.024	0.70
	Visit 7	80.67 ± 19.21	81.63 ± 20.77	87.37 ± 23.02	0.011	0.52
	Delta	4.27 ± 15.00	4.81 ± 15.52	6.54 ± 17.76	0.47	0.70
**LV end diastolic dimension (cm)**	n=	1361	1037	74		
	Visit 5	4.40 ± 0.48	4.37 ± 0.48	4.42 ± 0.54	0.06	0.99
	Visit 7	4.35 ± 0.49	4.28 ± 0.50	4.37 ± 0.56	0.82	0.12
	Delta	-0.05 ± 0.35	-0.09 ± 0.37	-0.04 ± 0.27	0.038	0.06
**Mean wall thickness (cm)**	n=	1366	1049	75		
	Visit 5	0.97 ± 0.13	0.98 ± 0.13	0.99 ± 0.12	0.21	0.08
	Visit 7	1.02 ± 0.15	1.03 ± 0.15	1.05 ± 0.13	0.004	0.82
	Delta	0.04 ± 0.11	0.06 ± 0.12	0.06 ± 0.12	0.039	0.05
**LVEF (%)**	n=	1300	980	71		
	Visit 5	65.65 ± 5.61	65.78 ± 5.62	65.60 ± 6.22	0.75	0.84
	Visit 7	63.27 ± 6.80	63.59 ± 7.03	62.55 ± 7.04	0.81	0.6
	Delta	-2.38 ± 6.37	-2.18 ± 6.29	-3.05 ± 5.97	0.61	0.47
**LA volume index (ml/m2)**	n=	1337	1015	71		
	Visit 5	24.66 ± 7.20	25.01 ± 7.46	27.13 ± 8.57	0.02	0.07
	Visit 7	27.16 ± 8.60	27.97 ± 9.21	30.53 ± 10.72	0.005	0.032
	Delta	2.50 ± 6.76	2.96 ± 6.99	3.40 ± 7.31	0.21	0.40
**e' lateral (cm/s)**	n=	1360	1045	76		
	Visit 5	7.33 ± 1.98	7.10 ± 2.00	6.95 ± 2.01	0.23	0.77
	Visit 7	6.69 ± 1.94	6.54 ± 2.00	6.81 ± 2.08	0.51	0.28
	Delta	-0.64 ± 2.07	-0.56 ± 2.11	-0.14 ± 2.22	0.08	0.20
**E/e' lateral (ratio)**	n=	1354	1041	76		
	Visit 5	9.43 ± 3.16	10.05 ± 3.64	10.48 ± 3.58	0.002	0.21
	Visit 7	11.75 ± 4.60	12.60 ± 4.95	12.37 ± 4.39	0.022	0.38
	Delta	2.32 ± 3.88	2.55 ± 4.23	1.89 ± 3.85	0.93	0.99
**E/A ratio**	n=	1287	982	70		
	Visit 5	0.87 ± 0.25	0.85 ± 0.25	0.83 ± 0.29	0.88	0.90
	Visit 7	0.86 ± 0.28	0.86 ± 0.30	0.85 ± 0.29	0.07	0.07
	Delta	-0.01 ± 0.27	0.01 ± 0.30	0.01 ± 0.30	0.045	0.046

Table S3: Echo measurements at Visit 5 and Visit 7 and their change, by frailty category at Visit 5 excluding participants with H_2FPEF score >6.

	Category	Robust	Pre-frail	Frail	p-value for trend	
					Model 1	Model 2
**LV mass index (g/m^2)**	n=	1341	1021	75		
	Visit 5	76.32 ± 17.23	76.83 ± 17.84	81.81 ± 21.84	0.008	0.52
	Visit 7	80.63 ± 19.23	81.53 ± 21.06	88.73 ± 24.61	0.005	0.45
	Delta	4.31 ± 15.00	4.71 ± 15.34	6.92 ± 18.46	0.53	0.80
**LV end diastolic dimension (cm)**	n=	1350	1031	77		
	Visit 5	4.40 ± 0.48	4.37 ± 0.49	4.42 ± 0.56	0.05	0.99
	Visit 7	4.35 ± 0.49	4.28 ± 0.51	4.38 ± 0.58	0.72	0.14
	Delta	-0.05 ± 0.35	-0.09 ± 0.37	-0.05 ± 0.28	0.048	0.06
**Mean wall thickness (cm)**	n=	n=1355	n=1043	n=78		
	Visit 5	0.97 ± 0.13	0.98 ± 0.13	0.99 ± 0.12	0.11	0.15
	Visit 7	1.02 ± 0.15	1.03 ± 0.15	1.06 ± 0.14	0.004	0.83
	Delta	0.05 ± 0.11	0.05 ± 0.12	0.07 ± 0.12	0.08	0.10
**LVEF (%)**	n=	1291	974	73		
	Visit 5	65.70 ± 5.51	65.78 ± 5.63	65.38 ± 7.14	0.54	0.68
	Visit 7	63.30 ± 6.86	63.59 ± 7.15	62.33 ± 7.61	0.98	0.73
	Delta	-2.40 ± 6.52	-2.19 ± 6.45	-3.05 ± 5.98	0.63	0.49
**LA volume index (ml/m2)**	n=	1328	1010	75		
	Visit 5	24.61 ± 7.18	24.91 ± 7.33	27.28 ± 8.16	0.012	0.07
	Visit 7	27.11 ± 8.85	27.85 ± 8.93	30.69 ± 10.59	0.005	0.06
	Delta	2.50 ± 6.91	2.94 ± 6.87	3.41 ± 7.26	0.31	0.58
**e' lateral (cm/s)**	n=	1350	1036	78		
	Visit 5	7.33 ± 1.96	7.12 ± 2.01	6.91 ± 1.95	0.32	0.87
	Visit 7	6.68 ± 1.92	6.54 ± 2.00	6.80 ± 2.09	0.38	0.22
	Delta	-0.65 ± 2.05	-0.57 ± 2.10	-0.10 ± 2.14	0.08	0.19
**E/e' lateral (ratio)**	n=	1344	1032	78		
	Visit 5	9.43 ± 3.16	10.02 ± 3.67	10.31 ± 3.22	0.005	0.39
	Visit 7	11.79 ± 4.63	12.59 ± 5.03	12.23 ± 4.12	0.048	0.63
	Delta	2.36 ± 3.88	2.57 ± 4.23	1.92 ± 3.89	0.98	0.89
**E/A ratio**	n=	1279	978	73		
	Visit 5	0.87 ± 0.24	0.85 ± 0.24	0.82 ± 0.28	0.78	0.81
	Visit 7	0.86 ± 0.28	0.86 ± 0.30	0.84 ± 0.26	0.12	0.11
	Delta	-0.01 ± 0.27	0.01 ± 0.30	0.02 ± 0.31	0.07	0.07

Table S4. Baseline characteristics at Visit 5 of the study population for the incident frailty analysis, overall and by subsequent frailty category at Visit 6.

Variable at V5	Overall at V5 (n= 1,648)	Frailty category at V6			p-value (trend)
		Robust (n=845)	Pre-frail (n=754)	Frail (n=49)	
Age, mean ± SD, years	73.6 ± 4.2	72.9 ± 3.9	74.2 ± 4.4	74.5 ± 4.1	< 0.001
Male, n (%)	755 (45.8%)	401 (47.5%)	331 (43.9%)	23 (46.9%)	0.24
Black, n (%)	333 (20.2%)	155 (18.3%)	164 (21.8%)	14 (28.6%)	0.029
Hypertension, n (%)	1126 (68.6%)	626 (75.4%)	582 (78.4%)	40 (85.1%)	0.06
Diabetes, n (%)	381 (23.1%)	168 (19.9%)	212 (28.1%)	25 (51.0%)	< 0.001
BMI, mean ± SD, kg/m^2	28.4 ± 5.1	27.9 ± 4.4	27.9 ± 5.4	30.4 ± 6.6	0.09
eGFR, mean ± SD, ml/min/1.73m^2	73.1 ± 15.2	67.1 ± 16.2	63.5 ± 17.7	55.4 ± 19.9	< 0.001
CHD, n (%)	171 (10.5%)	89 (10.7%)	111 (14.9%)	9 (18.8%)	0.006
MI, n (%)	127 (8.1 %)	46 (5.4 %)	79 (10.5%)	7 (14.3%)	< 0.001
Stroke, n (%)	32 (1.9 %)	23 (2.7 %)	34 (4.5 %)	3 (6.1 %)	0.033
Echocardiographic measurements					
LVMi	76.85 ± 17.89	75.5 ± 17.0	77.6 ± 18.1	87.4 ± 25.4	< 0.001
LVEDD	4.40 ± 0.49	4.4 ± 0.5	4.4 ± 0.5	4.5 ± 0.6	0.12
MWT	0.98 ± 0.13	1.0 ± 0.1	1.0 ± 0.1	1.1 ± 0.2	< 0.001
LAVi	24.98 ± 8.42	24.4 ± 6.9	25.6 ± 9.8	26.2 ± 7.4	0.003
e' lateral	7.22 ± 1.95	7.4 ± 1.9	7.1 ± 1.9	5.9 ± 1.7	< 0.001
E/e' lateral	9.60 ± 3.33	9.2 ± 3.1	9.9 ± 3.5	11.2 ± 4.0	< 0.001

E/A ratio	0.87 ± 0.25	0.9 ± 0.2	0.9 ± 0.3	0.8 ± 0.3	0.025

P values assess for trend for each variable across all categories. Data is shown as frequency and proportion or mean ± standard deviation. BMI= body mass index, eGFR= estimated glomerular filtration rate by CKD-EPI, CHD= coronary heart disease, MI= myocardial infarction, LVMi= left ventricular mass index, MWT= mean wall thickness, LVEDD= left ventricular end diastolic dimension, LAVi= left atrial volume index.

Table S5. Visit 5 characteristics of the population included for analysis 3. P-values compare participants included in the analysis to (1) participants alive but excluded from the analysis, and (2) participants who died between Visit 5 and Visit 6.

	Included in analysis	Alive Visit 6 but Excluded	P-value	Died Between V5 and V6	P-value
V5 Variable	n=1648	n=3653	(Included vs Alive at Visit 6 but excluded)	n=1237	(Included vs Died between V5 and V6)
Age	74 ± 4	76 ± 5	< 0.001	79 ± 6	< 0.001
Male	755 (46%)	1345 (37%)	< 0.001	593 (48%)	0.26
Black	333 (20%)	925 (25%)	< 0.001	285 (23%)	0.07
Hypertension	1126 (69%)	2746 (76%)	< 0.001	953 (80%)	< 0.001
Diabetes	381 (23%)	1241 (34%)	< 0.001	525 (42%)	< 0.001
BMI	28 ± 5	29 ± 6	< 0.001	28 ± 6	0.05
eGFR	73 ± 15	70 ± 17	< 0.001	62 ± 20	< 0.001
CHD	171 (11%)	507 (14%)	< 0.001	303 (25%)	< 0.001
MI	127 (8%)	423 (12%)	< 0.001	241 (21%)	< 0.001
Stroke	32 (2%)	154 (4%)	< 0.001	88 (7 %)	< 0.001

Figure S1. Associations of transitions in frailty status from Visit 5 to Visit 7 with concomitant changes in participants free of greater than moderate valvular disease. Plot demonstrates model beta coefficients and 95% CI for echocardiographic measurements by changes in frailty status. Model 1 adjusts for demographics (age, sex, race, and field center), and HR and BP at echo visits. Model 2 additionally adjusts comorbidities (BMI, history of smoking, prevalent CHD, eGFR, diabetes, hypertension, history of stroke, and prevalent AF) at V5. No change in frailty status between visits is taken as the reference value for each comparison (X line at 0). *Indicate significant at p <0.05. LAVI= left atrial volume index; LVEDD= left ventricular end diastolic dimension; LVMI= left ventricular mass index; LVEF= left ventricular mass index; MWT= mean wall thickness.

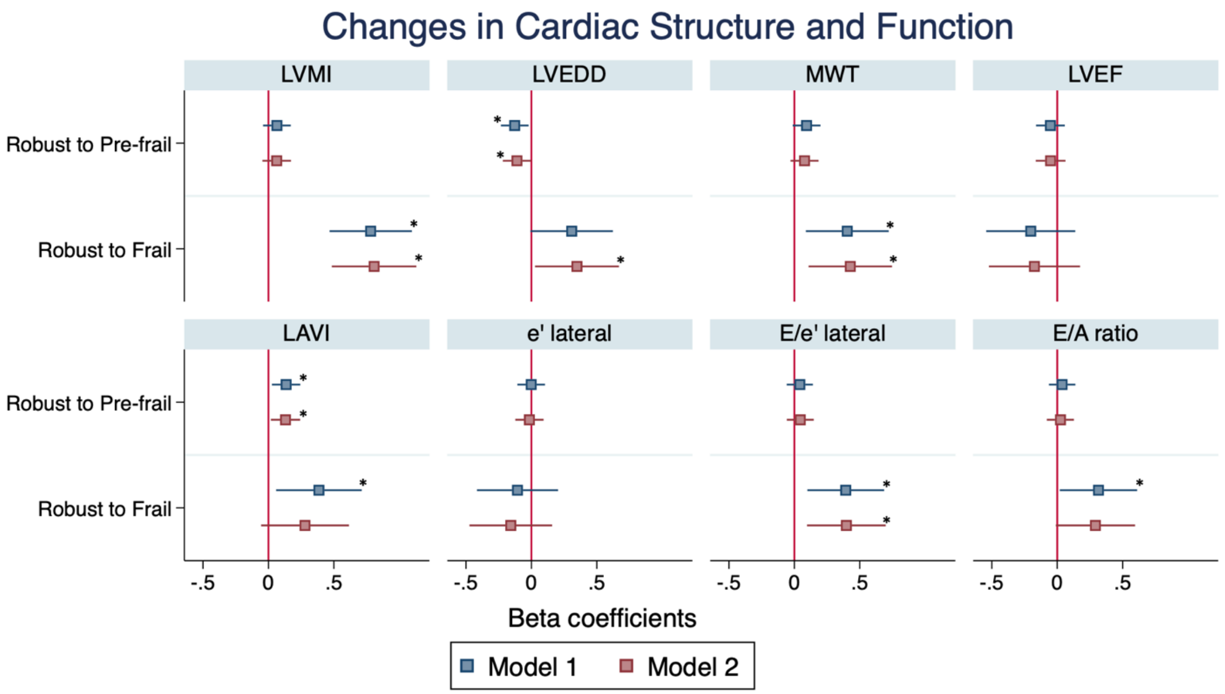

Figure S2. Associations of transitions in frailty status from Visit 5 to Visit 7 with concomitant changes excluding participants with moderate to severe dyspnea and NT-proBNP >450 pg/dl. Plot demonstrates model beta coefficients and 95% CI for echocardiographic measurements by changes in frailty status. Model 1 adjusts for demographics (age, sex, race, and field center), and HR and BP at echo visits. Model 2 additionally adjusts comorbidities (BMI, history of smoking, prevalent CHD, eGFR, diabetes, hypertension, history of stroke, and prevalent AF) at V5. No change in frailty status between visits is taken as the reference value for each comparison (X line at 0). *Indicate significant at p <0.05. LAVI= left atrial volume index; LVEDD= left ventricular end diastolic dimension; LVMI= left ventricular mass index; LVEF= left ventricular mass index; MWT= mean wall thickness.

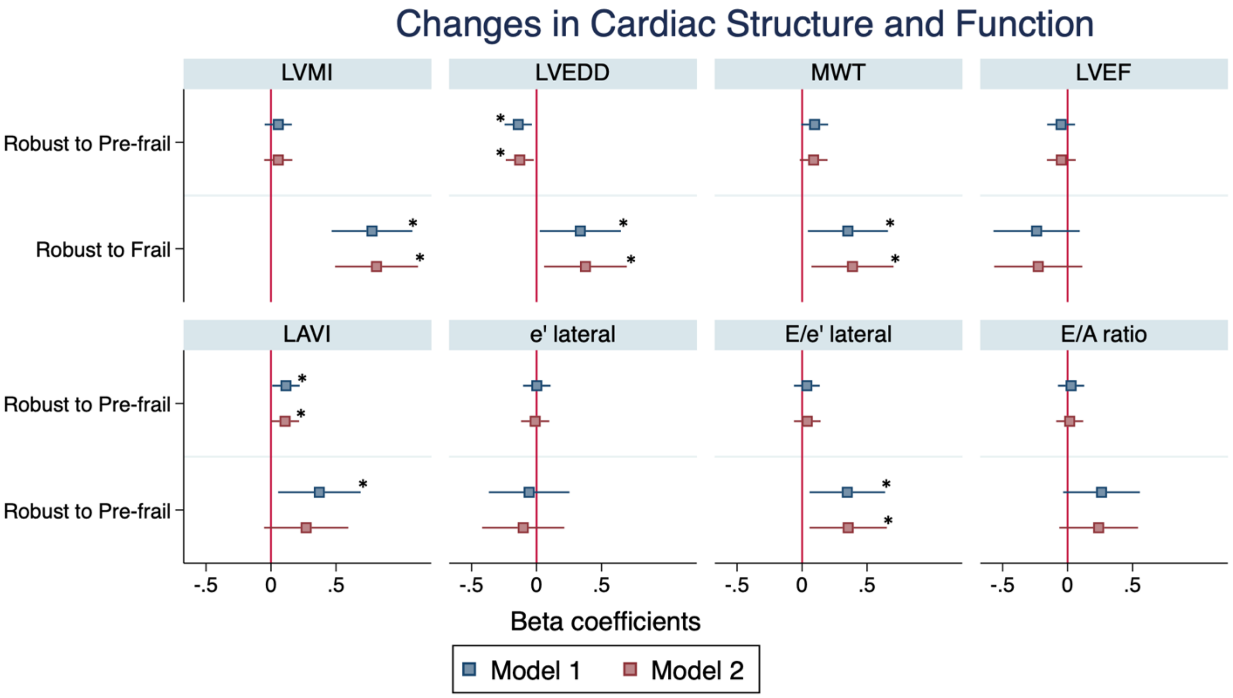

Figure S3. Associations of transitions in frailty status from Visit 5 to Visit 7 with concomitant changes excluding participants with a H_2FPEF score >6 (n=64). Plot demonstrates model beta coefficients and 95% CI for echocardiographic measurements by changes in frailty status. Model 1 adjusts for demographics (age, sex, race, and field center), and HR and BP at echo visits. Model 2 additionally adjusts comorbidities (BMI, history of smoking, prevalent CHD, eGFR, diabetes, hypertension, history of stroke, and prevalent AF) at V5. No change in frailty status between visits is taken as the reference value for each comparison (X line at 0). *Indicate significant at p <0.05. LAVI= left atrial volume index; LVEDD= left ventricular end diastolic dimension; LVMI= left ventricular mass index; LVEF= left ventricular mass index; MWT= mean wall thickness.

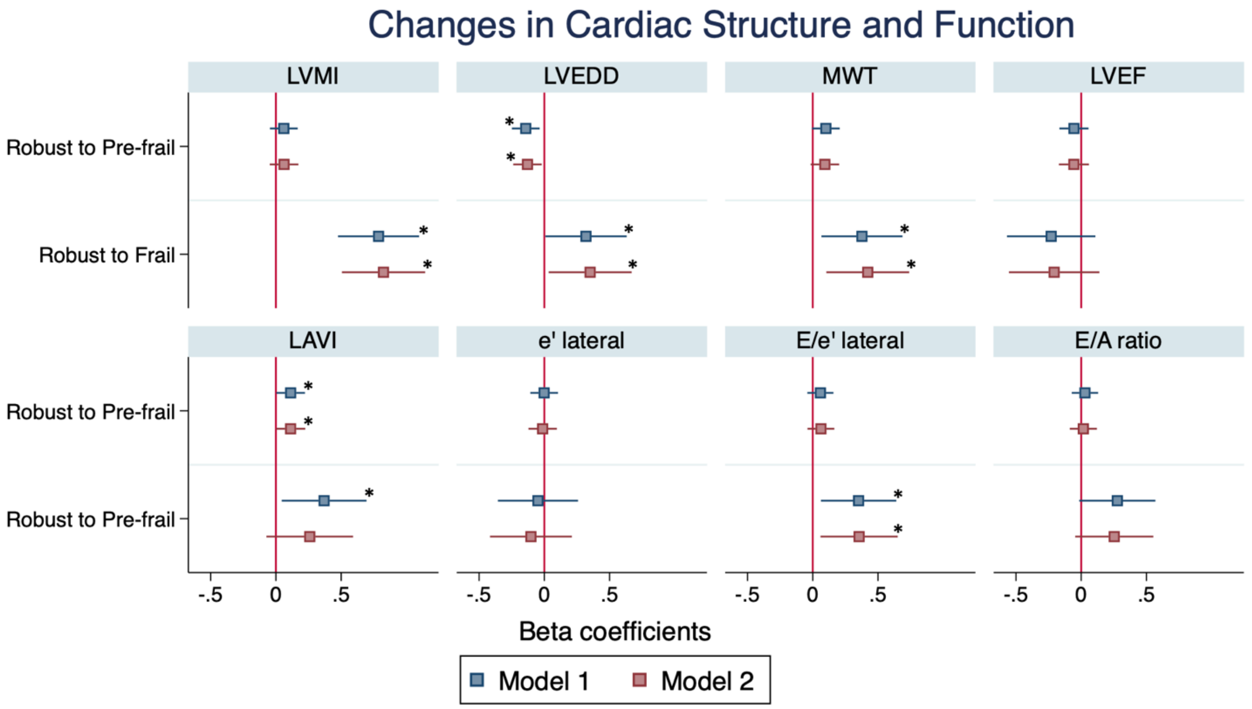

Figure S4: Association of measures of cardiac structure and function at Visit 5 with Incident Frailty (Panel A) or incident Pre-frailty (Panel B) at Visit 6. Model 1 adjusts for demographics (age, sex, race, and field center), and HR and BP at echo Visit 5. Model 2 additionally adjusts for comorbidities (BMI, history of smoking, prevalent CHD, eGFR, diabetes, hypertension, prevalent stroke, prevalent AF) at Visit 5. *Indicates odds associated with lower values.

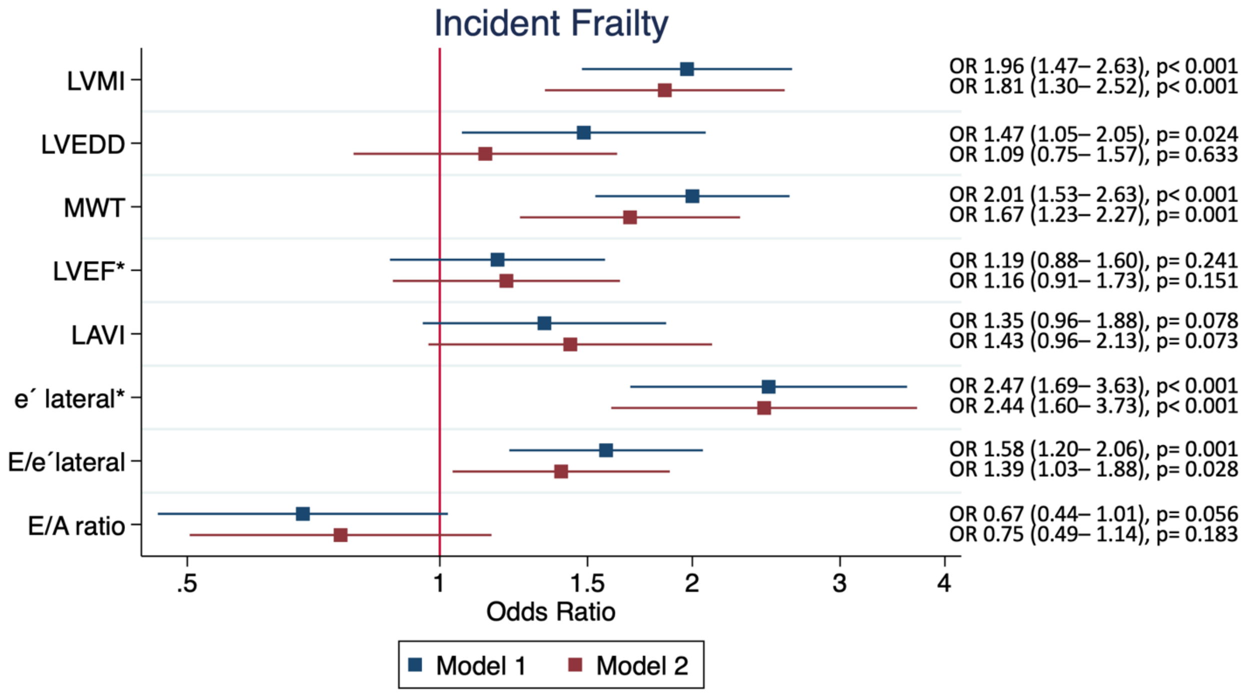

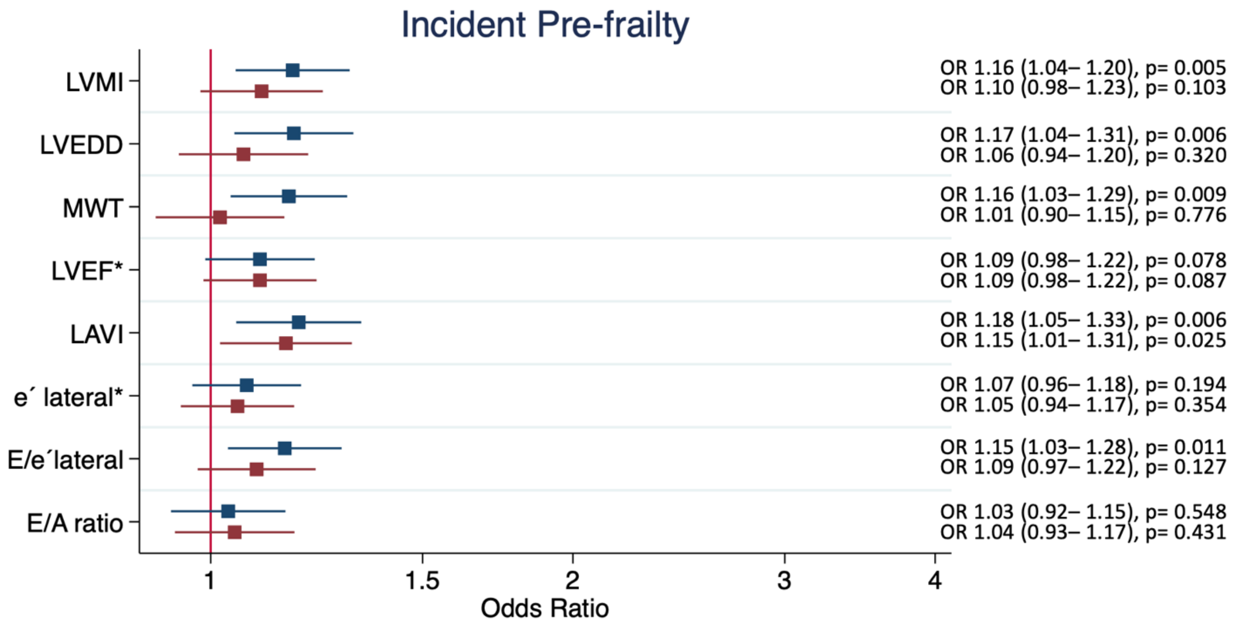
Incident Pre-frailty
LVMI
LVEDD
MWT
LVEF*
LAVI
e´ lateral*
E/e´lateral
E/A ratio
1
1.5
2
3
4
Odds Ratio
OR 1.16 (1.04– 1.20), p= 0.005
OR 1.10 (0.98– 1.23), p= 0.103
OR 1.17 (1.04– 1.31), p= 0.006
OR 1.06 (0.94– 1.20), p= 0.320
OR 1.16 (1.03– 1.29), p= 0.009
OR 1.01 (0.90– 1.15), p= 0.776
OR 1.09 (0.98– 1.22), p= 0.078
OR 1.09 (0.98– 1.22), p= 0.087
OR 1.18 (1.05– 1.33), p= 0.006
OR 1.15 (1.01– 1.31), p= 0.025
OR 1.07 (0.96– 1.18), p= 0.194
OR 1.05 (0.94– 1.17), p= 0.354
OR 1.15 (1.03– 1.28), p= 0.011
OR 1.09 (0.97– 1.22), p= 0.127
OR 1.03 (0.92– 1.15), p= 0.548
OR 1.04 (0.93– 1.17), p= 0.431

Figure S5: Association of measures of cardiac structure and function at Visit 5 with Incident Frailty by components at Visit 6. The model adjusts for comorbidities (BMI, history of smoking, prevalent CHD, eGFR, diabetes, hypertension, prevalent stroke, prevalent AF) at Visit 5. *Indicates odds associated with lower values. +Indicates statistical significance.

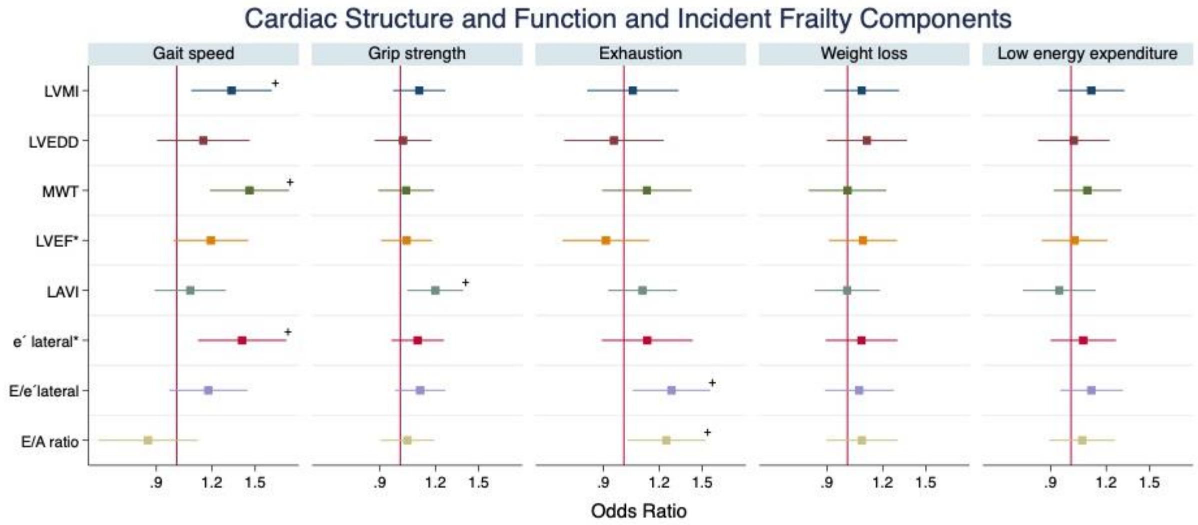

Figure S6: Association of measures of cardiac structure and function at Visit 5 with progression in frailty status among participants free of valvular heart disease. Model 1 adjusts for demographics (age, sex, race, and field center), and HR and BP at echo Visit 5. Model 2 additionally adjusts for comorbidities (BMI, history of smoking, prevalent CHD, eGFR, diabetes, hypertension, prevalent stroke, prevalent AF) at Visit 5. *Indicates odds associated with lower values.

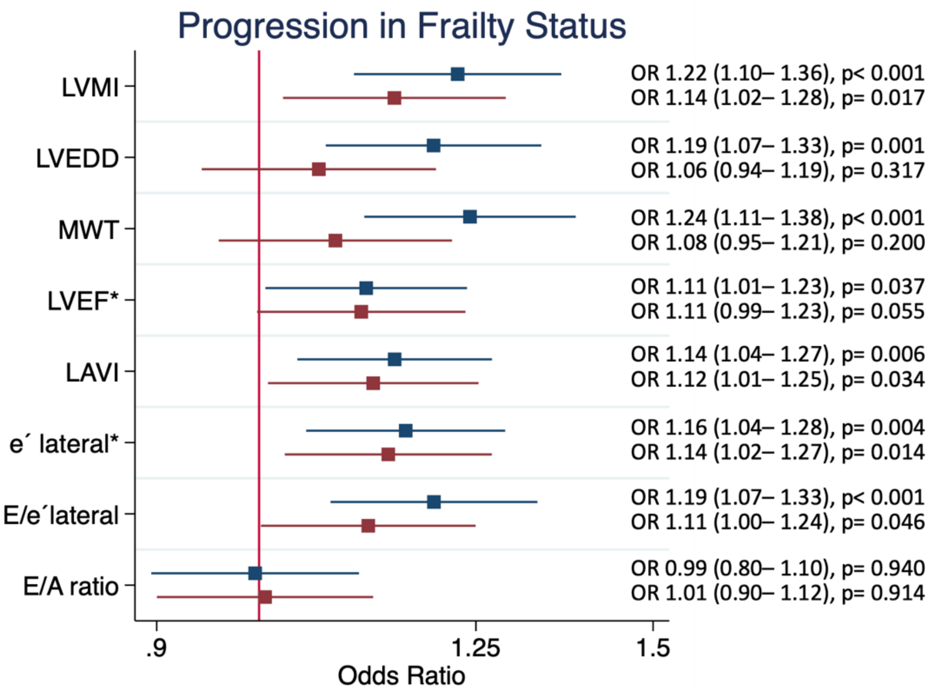

Figure S7: Association of measures of cardiac structure and function at Visit 5 with progression in frailty status among participants without moderate to severe dyspnea and NT-proBNP <450. Model 1 adjusts for demographics (age, sex, race, and field center), and HR and BP at echo Visit 5. Model 2 additionally adjusts for comorbidities (BMI, history of smoking, prevalent CHD, eGFR, diabetes, hypertension, prevalent stroke, prevalent AF) at Visit 5. *Indicates odds associated with lower values.

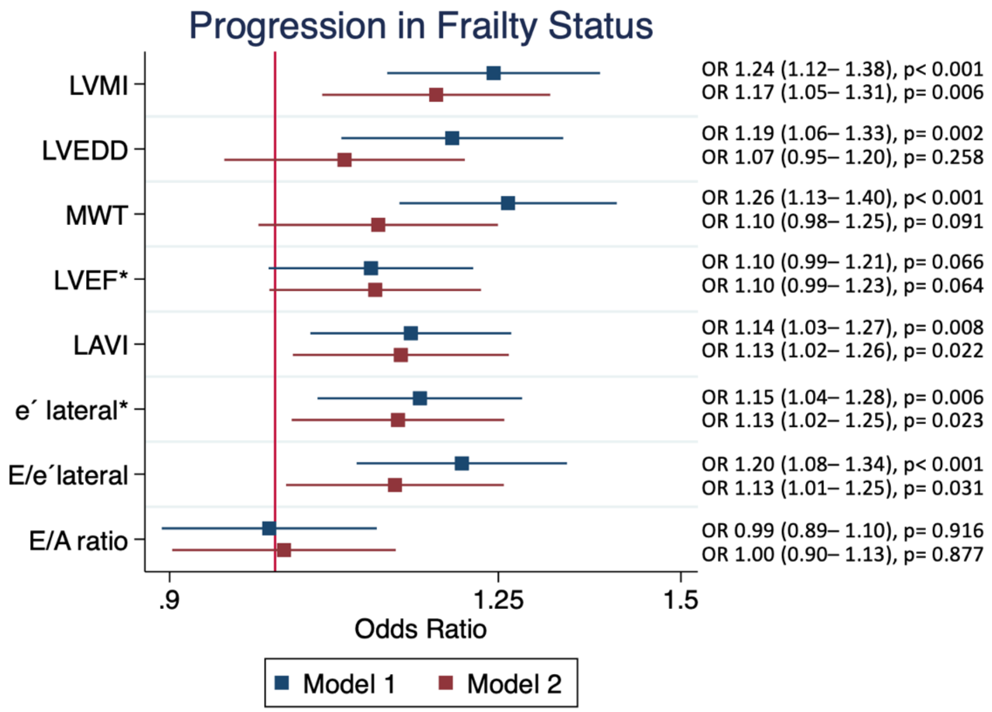

Figure S8: Association of measures of cardiac structure and function at Visit 5 with progression in frailty status among participants with a H_2FPEF score <6. Model 1 adjusts for demographics (age, sex, race, and field center), and HR and BP at echo Visit 5. Model 2 additionally adjusts for comorbidities (BMI, history of smoking, prevalent CHD, eGFR, diabetes, hypertension, prevalent stroke, prevalent AF) at Visit 5. *Indicates odds associated with lower values.

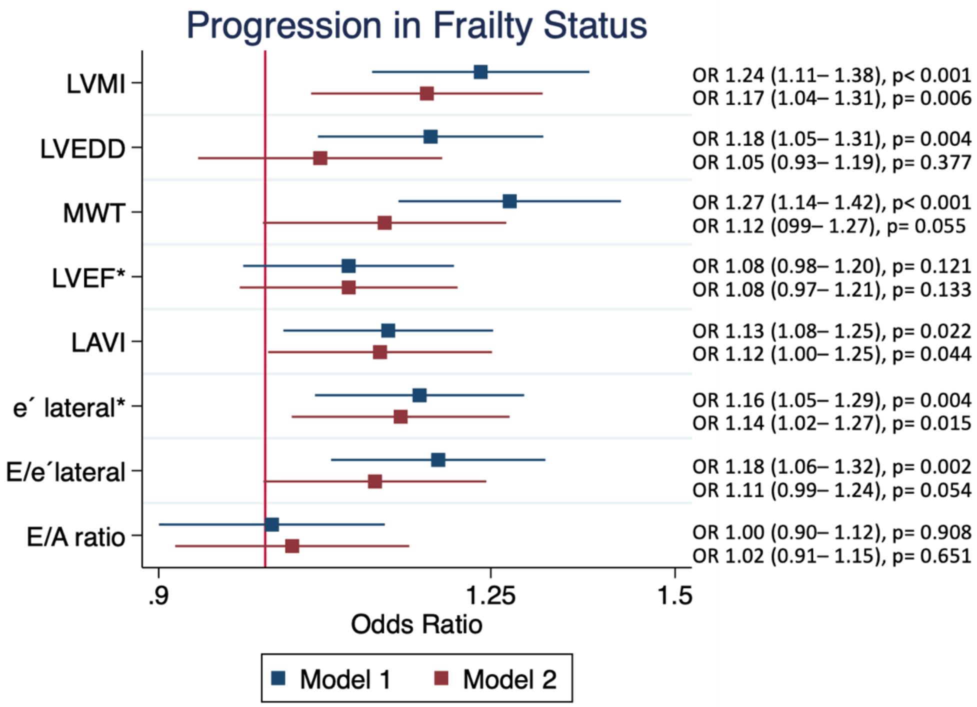

Supplement: Supplementary file 1 — Tables S1‐S5 Figures S1‐S8 [file JAH3-12-e029458-s001.pdf]
